# Multiomic analysis of human diabetic retinal disease reveals severity and region-specific impairment of the retinal neurovascular unit

**DOI:** 10.21203/rs.3.rs-11394372/v1

**Published:** 2026-10-30

**Authors:** Rachana Haliyur, Yang Shan, Nicholas J. Carruthers, Lynda Elghazi, Christopher Gates, Venkatesha Basrur, Felipe da Veiga Leprevost, Alexey I. Nesvizhskii, Patrice E Fort

**Affiliations:** 1 Department of Ophthalmology and Visual Sciences, Kellogg Eye Center, University of Michigan, Ann Arbor, MI; 2 Bioinformatics Core, University of Michigan, Ann Arbor, MI; 3 Department of Pathology, University of Michigan Medical School, Ann Arbor, MI; 4 Department of Computational Medicine and Bioinformatics, University of Michigan, Ann Arbor, MI; 5 Department of Molecular and Integrative Physiology, University of Michigan, Ann Arbor, MI

## Abstract

Diabetic retinal disease (DRD) is the leading cause of preventable blindness worldwide, but current therapeutic options only target vascular components in late-stage vision-threatening disease because the molecular mechanisms underlying early disease and affecting the whole retina are poorly understood. This study aimed to uncover disease-driving events of DRD using a multiomic approach to compare peripheral and central retinal tissue from human donors with diabetes without retinopathy or varying severity of diabetic retinal disease to their demographically matched non-diabetic counterparts. Integrative proteo-transcriptomic analysis combined with targeted validation reveals stage and region-specific disease drivers affecting the whole neurovascular unit defined by dysfunction in neuronal signaling, retinal metabolism, and neuroinflammation through activation of complement signaling. Further, our study reveals evidence of correlation of a subset of these retinal changes in the vitreous space in early disease, pointing to the specific role of this ocular fluid for DRD diagnosis and management. These findings are critical for the development of targeted therapeutic approaches to treat or prevent progression of DRD to vision threatening disease.

## Graphical Abstract

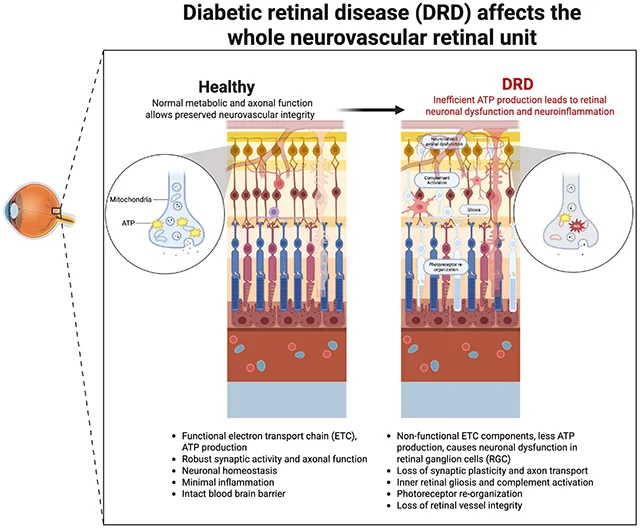

## INTRODUCTION

Diabetic retinal disease (DRD), traditionally thought of as a microvascular complication of diabetes mellitus (DM), leads to vision loss through retinal neurodegeneration associated with neuroinflammation and microvascular disruptions.^1^ Current therapeutic strategies for DRD, the leading cause of preventable blindness world-wide, target late-stage vision-threatening complications such as diabetic macular edema or proliferative diabetic retinopathy when vision is already compromised.^2–4^ Furthermore, existing treatment options are limited by visual recovery and high treatment burden. For this reason, identification of relevant clinical variables and biological targets prior to the development of vision loss is essential.

Traditional classification of diabetic retinopathy has relied exclusively on microvascular features visible on ophthalmic exam and/or diagnostic imaging. Early stages of DRD are characterized by microaneurysms, or outpouchings of capillary vessels, and progress to further vascular changes resulting from ischemia such as intraretinal hemorrhages and neovascularization (i.e. development of fragile new vessels) as seen in proliferative disease. While microvascular changes are important markers of disease progression, their presence indicates significant often irreparable neuroretinal injury. Retinal blood vessels intertwine with multiple retinal components including supporting cells, such as Müller glial cells, astrocytes, and the resident immune cells microglia, and a well-organized set of neurons. Animal and human tissue studies have discovered structural and functional changes occur within this neurovascular unit that coincide and even occur prior to the presentation of microvascular sequela.^5–8^ These and other studies have led to initiatives to revise the clinical and molecular classification and staging of DRD.^9–11^ However, to do this effectively, understanding the effects of DRD on the spatial and temporal changes within the neurovascular unit in clinically phenotyped human tissue is paramount.

To identify disease-driving events in DRD, we used a multiomic approach to integrate protein expression with gene expression within human retinal tissue from stages of diabetic retinopathy before the development of treatment requiring disease. Using retinal tissue obtained at the time of organ donation from patients with diabetes and/or DRD of varying severity, and their carefully matched non-diabetic counterparts, regional sample collection and analysis allowed both region-specific and stage-specific evaluation. Our analysis revealed multi-dimensional changes in the human retina including loss of neuroretinal function as evidenced by reduced synaptic re-modeling and neuronal function, gliosis, metabolic dysregulation, vascular disruption and extracellular matrix reorganization, and increased inflammation including complement activation. Critically, analysis of the corresponding vitreous revealed correlation and association with a subset of these retinal changes including a set of complement-specific protein changes reflected in early disease stages in this clinically accessible fluid. Further investigation into these neurodegenerative pathways expressed early in DRD prior to the development of overt microvascular damage or vision loss occur will allow identification of new targets for therapeutic management of DRD.

## METHODS

### Human participants

A total of 45 organ donors with diabetes and 21 organ donors without diabetes were used in this study. Donors with and without diabetes were identified by medical history with minimal ischemic time. De-identified medical histories provided information regarding diabetes type, duration, and treatment as well as demographic information and cause of death. The University of Michigan Institutional Review Board declared studies on de-identified human pancreatic specimens do not qualify as human subject research. Detailed information for donors can be found in Table 1, while specific information for donors included in the transcriptomic and proteomic datasets respectively are provided in Supplementary Tables 1–2.

### Human vitreous and retinal tissue sample collection

Human tissues were collected and preserved within 10 hours from cross clamp. Tissue from both eyes of each donor were received. One eye was flash frozen at time of recovery and processed for molecular analysis while the other was fixed in formalin and used for imaging analysis. Imaging of the fixed fellow eye with fundus photography and optical coherence tomography was used to confirm donor DRD severity grouping in conjunction with the de-identified clinical record. Few donors in the advanced group had evidence of early proliferative changes such as pre-retinal hemorrhage, however no included eyes had a documented history of vitrectomy, panretinal photocoagulation, or intravitreal injection.

### Proteomic analysis from human vitreous and retinal tissue using Mass Spectrometry TMT

Homogenized human vitreous and retinal tissues were depleted of high-abundance proteins using an Agilent Multiple Affinity Removal System (MARS) (Hu-6; Part #5188-5230, Agilent Technologies, Santa Clara, CA). Total protein was reduced with 5 mM DTT, alkylated with 15 mM 2-chloroacetamide, and precipitated using acetone. The resulting pellet was digested overnight at 37°C using a Trypsin/Lys-C mix. Peptides were labeled with TMT 10-plex reagents (Thermo Fisher Scientific, Waltham, MA) according to the manufacturer’s protocol and quenched with 5% hydroxylamine. The combined labeled samples were partitioned into eight fractions using a high-pH reversed-phase peptide fractionation kit (Thermo Fisher Scientific). Chromatographic separation was performed on an UltiMate 3000 RSLCnano UHPLC system (Thermo Fisher Scientific). Mass spectrometry was conducted on an Orbitrap Fusion Tribrid mass spectrometer (Thermo Fisher Scientific) using a multinotch-MS3 acquisition strategy to minimize reporter ion ratio distortion.^12^ Data analysis was performed in Proteome Discoverer (v2.4, Thermo Fisher Scientific), searching MS2 spectra against the SwissProt database with ≤ 1% FDR threshold. Quantitation was restricted to high-quality MS3 spectra exhibiting an average signal-to-noise ratio of at least 10 and < 50% isolation interference.

### Transcriptional analysis from human retinal tissue with RNA isolation followed by bulk RNA sequencing and qPCR

Total RNA was extracted from fresh-frozen retinal tissues using the RNeasy Plus Mini Kit (Qiagen, Germantown, MD) according to the vendor’s instructions. RNA integrity and concentration were assessed using the RNA ScreenTape assay on an Agilent Tapestation system (Agilent Technologies, Santa Clara, CA). Samples with RNA Integrity Number equivalent (RINe) > 7.0 were proceeded for downstream sequencing. Library construction and sequencing (150 pair end cycles) was carried out by University of Michigan Advanced Genomic Core on the Illumina NovaSeq-6000 platform. Total RNA was reverse transcribed into cDNA using a reverse transcription kit (NEBNext Ultra II RNA library prep kit, New England Biolabs, Ipswitch, MA) according to the manufacturer’s instructions. Quantitative PCR (qPCR) was performed using TaqMan Gene Expression Assays (Thermo Fisher Scientific, Waltham, MA) for the following targets: *C3* (Hs00163811_m1), *C4B* (Hs00416393_g1), and *HMOX1* (Hs01110250_m1). Target gene expression levels were normalized to β-actin (*ACTB*) as the endogenous internal control.

### Quality control measures for data integration and bioinformatic analysis

Data were prefiltered to remove genes with 0 counts in all samples. All RNA samples from one diabetic donor were removed from the analysis because the reported sex didn’t match with the expected gene expression profile for sex-specific genes.^13^ Samples were evaluated for known region-specific molecular markers to confirm spatial location. Further, the quality of the fovea extraction from other retinal regions was tested by PCA and marker gene expression. Specifically, foveal RNA samples were required to cluster with other fovea samples in PCA and to have higher expression of rod and cone marker proteins (*OPN1SW*, *OPN1LW*, *OPN1MW*, *CNGB3*, *PDE6H*, *LHX4*, *GNAT2*, *RHO*, *PDE6A*, *NR2E3*, *PPEF2*) relative to macula and peripheral samples. Three foveal transcriptomic samples, two from the non-diabetic group and one from the diabetes without DRD group, were excluded from the study for failing to meet these criteria.

### Transcriptomic data analysis

Downstream analysis of the RNA sequencing data was conducted in R (v4.1.1). Reads were mapped to GRCh38 using the STAR aligner.^14^ Count tables were generated using Rsubread.^15^ Differential expression was tested by limma (3.48.3) using a blocked design to account for the use of multiple samples from each subject.^16^ Contrasts were designed for (1) disease groups versus control with region as a covariate, (2) disease groups versus control within each region, and (3) interactions between disease and region effects. Covariates including age, gender, and procurement time were deemed to have a small impact on gene differential expression analysis and were not used. Gene t-statistics were submitted to the Search Tool for the Retrieval of Interacting Genes/Protein (STRING) (v12.0) functional enrichment tool, which uses Kolmogorov-Smirnov or aggregate fold-change tests to identify relevant gene categories.^17^ Pathways with FDR <0.5 were taken for further analysis. To reduce redundancy in visualized Gene Ontology (GO) categories, they were filtered to FDR <0.05 and clustered by Jaccard index for gene overlap using Euclidean distance and Ward’s method. The lowest FDR category for each cluster was selected for visualization. Gene and protein expression for each category was aggregated by taking the mean of the centered and scaled log-scale abundances for each region and disease severity of all its genes that were significant in any comparison (nominal p <0.05). Aggregated values were then normalized to have a mean of 0 and standard deviation of 1.

For line plots of the top interaction categories, aggregated expression values were calculated as described above and then shifted so non-diabetic (ND) samples aggregate abundance was 0. For evaluation of genes associated within a specific gene ontology process, genes with adjusted p< 0.05 and >±1.5 absolute fold change for any group relative to non-diabetic controls were selected for visualization. For bar plots, the bars indicate the mean abundance, and error bars are ±standard error of the mean (SEM). For box plots, samples were normalized to have the same total counts.

### Proteomic data analysis

Retinal proteomic data were analyzed similarly to the transcriptomic data with limma in a blocked design. Vitreous data were submitted to limma separately from retina without blocking or interaction effects as there was only vitreous sample per donor. Functional enrichment analysis was carried out using the STRING (v12.0) functional enrichment tool with redundant clusters removed in the same way.^17^

### Cell Type Deconvolution

Cell-type deconvolution of bulk RNA samples was carried out using AutoGeneS (v1.0.4).^18^ Single nuclei data were taken from Li et al. downloaded from the cellxgene atlas file (named e7e2207d-cdbf-4050-8378-02eb55d3948b.h5ad).^19^ Droplet “majorclass” was used to identify cell types. Several cell type pairs were considered indistinguishable and merged: amacrine cells with horizontal cells, rod cells with cone cells, and Müller glial cells with astrocytes. Retinal pigment epithelium cells were removed from the dataset. The top 4000 most variable genes were submitted to AutoGeneS, which was set to identify 400 genes to be used for deconvolution. Raw counts were submitted for deconvolution using the non-negative least squares algorithm. Deconvolution was also run using MuSIC (v1.0.0) using the same single cell reference.^20^

Differential expression models for each region were fit with either no cell-type covariates, bipolar cells (BC), retinal ganglion cells (RGC), Müller glial cells and/or astrocytes (MG/Astrocyte), and microglia together or on their own. Photoreceptor proportion was not included in the joint model to avoid having the proportions sum to one. The number of genes differentially expressed between advanced DRD samples and controls (q<0.1) were counted to assess covariate impact. T-statistics from selected models were submitted to functional enrichment analysis using STRING (v12.0) functional enrichment tool.^17^

### Western Blot Analysis

Fresh-frozen retinal tissue fragments from the macula or the periphery of 8 donors (n=8) of each group (ND: Non-diabetic, D: Diabetic, DRm: mild Diabetic Retinopathy, and DRad: advanced Diabetic Retinopathy) were sonicated in RIPA buffer (Thermo Fisher, Waltham, MA), and protein concentration was determined using a colorimetric protein assay (Bio-Rad, Hercules, CA). Total protein (25 ug per sample) was separated via SDS-PAGE using 4-12% Bis-Tris gels (Invitrogen/Thermo Fisher, Waltham, MA) and transferred to nitrocellulose membranes. Following blocking with the Everyblot blocking buffer (Bio-Rad, Hercules, CA), membranes were incubated overnight at 4°C with gentle agitation with the following primary antibodies: Actin (1:5000; #3700; Cell Signaling Technologies, Danvers, MA), Beta-2-Microglobulin (1:1000; # 13511-1-AP; Proteintech, Rosemont, IL), Synaptogamin-1 (1:1000; # 14511-1-AP; Proteintech, Rosemont, IL), IDH3A (1:1000; # 15909-1-AP; Proteintech, Rosemont, IL). Near-infrared fluorescent-labeled secondary antibodies from Jackson ImmunoResearch [JIR] were used (1:10000; #11-655-152, #11-625-150; JIR, West Grove, PA). Protein bands were visualized using the Azure 600 imaging system (Azure Biosystems, Dublin, CA). Densitometric analysis was performed using ImageJ, with target band intensities normalized to the loading control (β-actin). Data were analyzed and plotted using GraphPad Prism (v10.5, GraphPad, San Diego, CA); statistical significance was determined using a one-way ANOVA followed by Tukey’s post hoc test.

### Enzyme-Linked Immunosorbent Assay (ELISA)

Fresh-frozen retinal tissue fragments from the macula and the periphery of Non-diabetic (n=14-15), diabetic without retinal disease (D; n=15-16) or diabetic with diabetic retinal disease (DRD; n=15-16) donors were homogenized on ice in cold phosphate-buffered saline containing a broad-spectrum protease-inhibitor cocktail. Homogenates were sonicated, centrifuged and the clarified supernatants were collected without disturbing the pellet. Aliquots were stored at −80°C and subjected to no more than one freeze–thaw cycle. Total protein concentration was determined. Soluble terminal complement complex (sC5b-9) was quantified using the MicroVue SC5b-9 Plus EIA kit (QuidelOrtho, #A020) according to the manufacturer’s instructions. Samples were diluted in the kit sample diluent so that measured values fell within the standard-curve range. Kit standards, controls, retinal homogenates, and extraction-buffer blanks were analyzed in duplicate. Briefly, diluted specimens and standards were added to the antibody-coated microplate and incubated for the time and temperature specified in the instructions. Wells were washed using the supplied wash solution, incubated with the enzyme-conjugated detection reagent, washed again, and developed with the supplied chromogenic substrate. After addition of stop solution, absorbance was measured at 450 nm using a microplate reader. Concentrations were calculated from the standard curve and corrected for sample dilution. Results were normalized to total retinal protein and reported as sC5b-9 units per milligram of protein.

### Immunohistochemistry

For immunohistochemical detection, 10 μm thick frozen retinal sections from 3 independent (n=3) non-diabetic (ND) donors and their age-, sex- and race-matched diabetic donors with diabetic retinopathy (DR) were re-hydrated in PBS and permeabilized for 15 min in PBS with 0.3% Triton X-100. Sections were then incubated in blocking solution (3% donkey serum, 1% BSA in PBS) for 30 min at room temperature before incubating overnight at 4 C in the following primary antibodies and dilutions: rabbit anti-Beta-2-Microglobulin (1:500; # 13511-1-AP; Proteintech, Rosemont, IL), rabbit anti-SNAP25 (1:600; # 14903-1-AP; Proteintech, Rosemont, IL), rabbit anti-Synaptogamin-1 (Syt1) (1:400; # 14511-1-AP; Proteintech, Rosemont, IL), goat anti-collagen IV (1:1000; #1340-01; Southern Biotech, Birmingham, AL), mouse anti-glutamine synthetase (GS) (1:200; #610518; BD Bioscience, San Jose, CA), rat anti-glial fibrillary acidic protein (GFAP) (1:600; #13-0300; Invitrogen/Thermo Fisher, Waltham, MA), chicken anti-neurofilament H (NFH) (1:800; PA1-10002; Invitrogen/Thermo Fisher, Waltham, MA), Ulex Europaeus Agglutinin (UEA-1) Atto-594 (1:500; #78873; Sigma, Saint-Louis, MO). Sections were incubated with the appropriate secondary antibodies conjugated to Alexa Fluor dyes, AF488 or AF647 (Life Technologies). Nuclei were counterstained with Hoechst (Life Technologies). Negative controls omitted the primary antibodies. Images were acquired with a confocal microscope (Leica Stellaris 8 Falcon system, Wetzlar, Germany) Images were captured using the Leica Application Suite (LAS).

## RESULTS

### Clinical evaluation and molecular clustering of human donors along progression to DRD

To gain insight into the pathways driving retinal dysfunction, we focused on early stages of diabetic retinal disease (DRD) by collecting retinal tissue from deceased human donors with diabetes (diabetes, n=23 donors, aged 69.3±4.6 years, hemoglobin A1C (HbA1C) 6.9±1.6%), mild non-proliferative diabetic retinopathy (mild DRD, n=11 donors, aged 67.5±5.8 years, HbA1C 8.3±1.4%), and severe non-proliferative diabetic retinopathy or early proliferative changes (advanced DRD, n=11 donors, aged 65.3±8.4, HbA1C 8.9±2.0%) compared to age-matched non-diabetic controls (ND, n=21 donors, aged 69±4.7 years) (Figure 1a–b). Clinical phenotyping was obtained from available donor records and DRD severity grading was confirmed with postmortem evaluation using fundus photography and optical coherence tomography of the fellow eye. Clinical metrics of diabetes, such as average HbA1C and diabetes duration, corresponded with expected DRD severity (Table 1 and Figure 1c–d). Principal component analysis (PCA) of samples from each disease cohort showed significant overlap between groups with close clustering of the samples without diabetes and the advanced DRD state diverging most distinctly from other samples both by RNA and protein in the regions investigated (Figure S1a–f).

### Identification of biological processes associated with DRD severity

We next investigated differentially expressed gene products identified by DRD severity compared to human donors without diabetes with region as a co-variate. Respectively, 2928 genes and 26 proteins were significantly differentially expressed (p<0.01) between those with diabetes without DRD compared to ND, 3396 genes and 193 proteins between those with mild DRD compared to ND, and 5610 genes and 701 proteins between advanced (adv) DRD compared to ND (Figure 1e). This distribution was similar when comparing differentially expressed genes and proteins by false discovery rate (FDR <0.1) (Figure S1g). Significant differentially expressed gene products were slightly greater in the posterior pole (fovea and macula for RNA and macula for protein) when compared to the peripheral retina and the number increased with disease severity (Figure 1f–g and Figure S1h–i). Functional enrichment analysis of gene product differential expression was used to identify the main biological processes affected by disease (Figure 1h–m). Biological processes associated with cellular respiration, mitochondrial activity, and metabolism were significant in both proteomic and transcriptomic datasets in advanced DRD (Figure 1l–m). Changes in processes associated with mitochondrial function occur in mild disease (Figure 1j) and even prior to clinical evidence of DRD (Figure 1i). Pathways related to neurotransmitter transport and synaptic activity were similarly enriched in the advanced stage (Figure 1l) with evidence of these changes detected even before clinical DRD by transcriptomics (Figure 1h). Also indicative of the global effect of diabetes on retinal physiology, gene expression modulation through chromatin remodeling and mRNA processing were among the main processes identified and enriched in the mild DRD stage by proteomic analysis (Figure 1k).

To visualize the dynamics of how DRD affected these processes across disease severity, we plotted aggregated gene expression levels for significant pathways for transcriptomic (Figure 1n) and proteomic (Figure 1o) data. Pathways associated with mitochondrial activity, such as ATP synthesis, and neuronal signaling, such as synaptic dynamics, decreased with increasing disease severity in all regions but was most pronounced in the fovea (Figure 1n **clusters 1 and 3**). The significance of these findings is reflected by the same pattern change in these pathways within the proteomic data (Figure 1o **cluster 4**). Pathways involved in response to exogenous stimuli, including antigen processing and presentation, demonstrated a different pattern and were first relatively unchanged in early stages until being significantly increased in advanced DRD in all regions (Figures 1n **cluster 4** and 1o **cluster 3**). Pathways associated with protein synthesis had a profile of maximal enrichment in samples with diabetes without DRD in all studied regions (Figure 1n **cluster 6**). Genes in categories related to histone modification and photoreceptor function increased with disease severity, with this pattern particularly localized to the fovea compared to the macula and periphery by transcriptomic data (Figure 1n **cluster 7**).

### Retinal biological processes in DRD are affected in a region-specific manner

To identify DRD-related region-specific changes, pathways that were enriched in genes with strong interaction effects by region (i.e. fovea, macula or periphery) were identified (Figures 2a and 2f). Upset plots were used to characterize the overlap of biological processes affected by disease stage both retina-wide and by region (adjusted p-value <0.05) (Figure 2b and 2g). Most pathways were identified by retinal transcriptomics when comparing advanced DRD to ND (254) while retinal proteomic data revealed many pathways in both the mild DRD to ND (267) and advanced DRD to ND (229) comparisons. In the transcriptomic and proteomic datasets, many processes were distinct between retina-wide and region-specific interaction analyses (Figure 2b and 2g) suggesting the region-affected pathways are not merely a subset of the retina-wide analysis.

Evidence for retinal photoreceptor re-organization is seen by an initial increase in microtubule and cilia activity in DM without DRD (“Diabetic”) followed by decreased expression of these pathways with DRD (Figures 2a and 2c **panel 1**). Similarly, increased expression of photoreceptor cell function and maintenance genes was also primarily observed in DM without DRD, particularly in the central retina (fovea and macula; Figure 2c **panel 2**). Changes seen in metabolic function were more nuanced. Decreased expression of lipid biosynthesis genes localized to the central retina (fovea and macula) in advanced DRD by transcriptomic data (Figure 2a and 2c **panel 4**), while the timing and pattern of changes in aerobic respiration varied as a function of region. Decreased oxidative phosphorylation was first detected in the fovea even in the absence of DRD prior to being observed throughout the retina with advancing disease (Figure 1n **clusters 1** and **2** and Figure 2c **panel 5**), while proton transport through the electron transport chain increased at later stages and specifically in the fovea (Figure 2c **panel 7**). Increased inflammation with DRD stage were most highly detected within the macula and periphery by both RNA and protein (Figures 1n–o, 2c **panel 6,** and 2h **panel 3**) but did not appear as prominently in the fovea (Figure 2c **panel 6**). Gene regulatory changes, both pre- and post-translational, were most prominent in mild DRD and decreased expression for the identified pathways localized to the periphery (Figures 2a, 2c **panel 3,** and 2f).

To assess how retinal findings are reflected within the adjacent and more clinically accessible vitreous humor, we compared the proteomic analysis of the vitreous compartment from the same donors used for retinal analysis in each DRD stage. Pathway analysis revealed that the humoral immune response, blood coagulation, and complement were upregulated in the vitreous of donors with mild and advanced DRD (Figures 1o **cluster 3** and 2f and Figure S2a). Further comparison of the patterns identified within the retina to the vitreous space revealed increased expression of inflammatory processes (Figures 1o **cluster 3** and 2f) and decreased synaptic and metabolic function (Figure 1o **cluster 4** and Figure S2b–c) followed a similar directionality to the retina with disease progression. Importantly, expression of pathways associated with cytoskeleton organization decreased with disease severity in the vitreous while it increased in the macula and periphery of the retina (Figures 1o **cluster 1** and 2f) highlighting the non-linear and systematic nature of the composition of this fluid and the adjacent retinal tissue.

While mitochondrial function seemed globally affected across all regions with increasing disease severity, we set out to define the region specificity of aerobic respiration changes (Figure 2c) by plotting the genes identified with the largest interaction effect between region and disease group (Figure 2d and 2e). Decreased expression of proteins within the electron transport chain (ETC) important in establishing a protein gradient for ATP synthesis were observed with advancing disease severity, especially in the fovea. Detailed analysis of this effect highlighted genes involved in ETC Complex I (*NDUF* family proteins), which are responsible for oxidation of NADH to NAD+, *UQCRHL*, the electron transfer reaction between cytochrome c1 and c, cytochrome c (*CYCS*), and ATP Synthase membrane unit (*ATP5ME*). Interestingly, this was coupled with increased expression of NADH dehydrogenase proteins (MT-ND4 and MT-ND5), which are also associated with ETC Complex I and important in ETC function as variants in these genes are associated with Leber’s hereditary optic neuropathy (LHON).^22–24^ We further compared the expression profiles of these identified genes to the retinal proteomic data (Figure 2i), which confirmed a primary effect of diabetes on this pathway in the posterior pole with decreases in proteins such as NDUFB1 in advanced DRD in this region as was seen at the transcript level.

Our analysis further identified decreased cholesterol biosynthesis as a foveal-specific process (Figures 1n **group 1** and 2a), driven by decreased expression of proteins involved in cholesterol homeostasis and endoplasmic reticular response to oxidative stress (Figure S2d). Decreased function of *DHCR24* may lead to desmosterol accumulation, associated with neurodegeneration and decreased endothelial protection.^25,26^ Finally, while not identified as a region-specific change, we further investigated the identified global decrease in synaptic function (Figure 1n–o). We found expression of proteins critical for the structural scaffold (i.e. BSN, SYN1) and function (NRXN1, PRKAR1B, RAB3B) of neuronal synapses were decreased in both the transcriptomic and proteomic data (Figure S2e–f). These gene changes were even reflected within the vitreous for NRXN1 and SYN1 (Figure S2g).

### Human DRD impacts the whole neuroglial retinal unit

To further understand the primary cellular drivers of the described changes seen in bulk RNA sequencing, we performed cell-type deconvolution of the bulk transcriptomic analysis based on single cell and nucleus profiling from published data, which notably did not include cells associated with the retinal vasculature limiting analysis to retinal neuronal and non-vascular supportive cells.^19^ Retinal cell types localized as expected by region, with retinal ganglion cells particularly represented within the foveal samples reflecting the peak ganglion cell density in the perifovea (Figure 3a). In the transcriptomic dataset, the deconvolution data revealed an increase in the relative proportion of supporting cells, specifically astrocytes and Müller glia, with increased DRD severity in all regions supportive of increased gliosis and inflammation identified in our analysis (p<0.05 β regression). A similar trend was observed in the proteomic data. We also found a progressive reduction in the proportion of foveal retinal ganglion cells with advanced DRD, consistent with neurodegeneration (p<0.05 β regression). Next, we investigated the enrichment between cell-specific markers taken from the public single cell retina data and significantly affected proteins in each disease stage by region (Figure 3b). Of note, the relative contribution to the retinal proteome was much more biased towards supporting cells, both microglia and Müller glia/astrocytes, compared to the transcriptomic data.

We next used co-variate analysis for each cell-type to determine whether DRD-associated transcriptional changes could be ascribed to changes in cell-type abundance. Each cell-type was introduced into the model for each disease stage and region to identify when genes were more closely associated with the cell-type than the disease. Disease-specific differentially expressed genes in advanced DRD appeared to be strongly driven by retinal ganglion cells in the fovea and by Müller glia and astrocytes in the macula (Figure 3c). Changes in gene expression localized slightly more to microglia in the periphery, but this cell type appeared to play a significant role in all regions (Figures 3c and S3a). We further investigated the specific pathways represented in each cell-type, which confirmed cell specificity but also revealed region and cell-specific functional changes. In advanced DRD, pathways relative to phototransduction and protein synthesis were associated with photoreceptors in all retinal regions, but interestingly pathways involved in cellular respiration and neurotransmission associated to foveal RGCs and not photoreceptors (Figures 3d and S3a–c). Macular Müller cells and astrocytes were associated with matrix regulation, immune cell response, and regulation of angiogenesis (Figure 3e). Finally, as anticipated for resident retinal immune cells, peripheral microglia associated with immune cell localization and response (Figures 3f and S3d).

### Correlation of biological pathways identified by retinal transcriptomic and proteomic analysis

To further highlight DRD drivers, we next focused on pathways found in the retina by both transcriptomic and proteomic datasets. When comparing the GO biological processes (GOBP) identified between the retinal protein and RNA, decreased aerobic metabolism and neuronal processes, along with increased immune response correlated with advanced DRD (Figure 4a). This directional pattern of pathway changes is evident even when plotted in comparisons of diabetics without DRD and mild DRD with the most striking association between the RNA in mild DRD and the proteins in advanced DRD, supportive of a progressive notion of the disease process (Figure 4c). Identification of processes similar and unique to the vitreous were further highlighted by comparison of retinal protein to the vitreous protein dataset (Figure 4b and 4d). Immune activity, namely complement activation, could be appreciated uniquely in the vitreous in those with diabetes without DRD before similar patterns emerge in the retina with increasing disease severity (Figure 4b’). Interestingly, pathways such as mRNA processing, localization and splicing had increased expression with disease severity within the vitreous in contrast to the retinal macula and periphery, confirming pathway enrichment and directionality unique to the vitreous compartment (Figures 4b’ and S4a–c).

When comparing advanced retinal DRD to ND, there were 222 enriched GOBP identified only by proteomic analysis and 206 identified only by transcriptomic analysis while 39 pathways overlapped (Figure 4e). The pathways associated with decreased synaptic function and immune activation were identified in both datasets when comparing advanced DRD to ND (Figure 4f **clusters 1**, **2** and **3**) while decreased expression of pathways associated with mitochondrial function and cellular metabolism was enriched in both mild DRD and advanced DRD when comparing to ND (Figure 4f **cluster 4**), suggesting that these latter pathway changes occur earlier in the progression of the disease. We then looked at correlation of gene products differentially expressed between the vitreous protein and either retinal RNA (Figure S4d) or protein (Figure S4e). The top correlating genes between the retinal and vitreous datasets correspond to mitochondrial metabolic function, complement and iron homeostasis (Figure 4g), further confirming reflection of significant retinal findings within the vitreous compartment.

### Functional markers of decreased metabolic and neuronal function are identified in advancing macular DRD

To define and characterize how DRD affects metabolic function and synaptic activity within the human retina as identified by multiomic analysis, we used pathway-specific testing to identify nodes and candidate proteins, which were then confirmed by orthogonal methodologies. Specific extraction of data relative to pathways associated with mitochondrial and metabolic function using expression levels of top genes in the transcriptomic (Figure 5a) and proteomic (Figure 5b) analyses confirmed overall decreased metabolic function within all retinal regions. While both datasets support a progressive reduction in aerobic respiration in the retina, this finding in mitochondrial function was not detected in the vitreous (Figures 5a–b and S5a–b). Mitochondrial Krebs cycle functional enzyme isocitrate dehydrogenase 3 alpha (IDH3A) was among the proteins significantly decreased by proteomic analysis (Figure S5b), a finding confirmed in advanced DRD retinal samples by western blot analysis in both the periphery (PR) and macula (MAC) (Figures 5c and S5c). Cellular localization of reduced IDH3A expression was further defined by immunofluorescence analysis of retinal cross-sections from donors with advanced DRD when compared to tissues from matched non-diabetic donors (Figures 5d and S6a). Interestingly, this reduction was particularly striking in the inner retina, consistent with the cell deconvolution data that revealed a central impact of diabetes on cellular respiration in retinal ganglion cells (Figure 3d).

We next focused on proteins crucial to neuronal and synaptic function. Targeted pathway analysis highlighted decreased expression of genes (Figure 5e–f) and proteins (Figure 5g–h) associated with regulation of neurotransmitter secretion. Protein levels for both (Synaptotagmin-1, SYT1) (Figures 5i, k and S5d, f–g) and Synaptosomal-Associated Protein, 25kDa (SNAP-25) (Figure S6b–c) were significantly decreased in retinal tissues from donors with advanced DRD. Immunofluorescence analysis further demonstrated that decreased expression of these proteins in DRD samples was localized to plexiform layers and particularly reduced in the inner plexiform layer (Figures 5i, S5g, and S6b–c). SNAP25 had a dramatic reduction in signal within the retinal nerve fiber layer consistent with reduced expression for the retinal ganglion cell axonal marker neurofilament protein H (NFH, Figure S6b–c). This finding coincided with increased glial activation as demonstrated by glial fibrillary associated protein (GFAP) immunoreactivity (Figures 5i and S6b) and is consistent with retinal stress seen in advanced neurodegeneration.

We then sought to integrate these findings within the overall neurovascular unit by assessing the local retinal vascular integrity. While the vascular extracellular matrix visualized by collagen IV staining remained conserved in tissues from advanced DRD donors, signal for Ulex europaeus agglutinin-1 (UEA), a lectin specifically interacting with endothelial cells, was almost completely lost in these regions, confirming the presence of clinical vascular changes, namely reduced or loss of perfusion, that have long characterized DRD (Figures S5g and S6c). While these findings coincided with described neuronal dysfunction, these vascular changes also correlated with increased levels of basement membrane marker beta-2 microglobulin (B2M) in the retina from donors with advanced DRD. This increased B2M localized around the retinal vasculature in DRD, which was most noticeable around vessel structures within the inner retina where there was concurrent increased expression of gliotic marker GFAP (Figures 5j and S5h). Together these findings of overt gliosis, decreased neuronal function, and decreased vessel integrity highlight the impact of diabetes on the entire neurovascular unit in advanced DRD and raised the question regarding the role of neuroinflammation.

### Innate immune response activation correlates with DRD severity in a region-specific manner

Pathway analysis of both transcriptomic (Figure 2a and 2c) and proteomic (Figure 2f and 2h) datasets unveiled a potential association of DRD progression with multiple pathways involved in inflammation and immune response. Further analysis revealed that immune surveillance and activation, specifically the complement system of the innate immunity, was enriched at both the transcript (Figure 6a–b) and protein (Figure 6c–d) level with increasing DRD severity. Of note, this included proteins of the classical (C1R, C1RL) and alternative (CFI, CFB, CFH) pathways and was observed in all regions of the retina and vitreous as a function of DRD progression (Figure 6a–d). To better understand the role of complement activation in DRD pathophysiology, we performed validation by qPCR and ELISA, which confirmed increased expression of key complement components including the final cytolytic complement membrane attack complex (MAC) (Figure 6e–k). Immunofluorescence demonstrated the increasing presence of the MAC complex and localized it around vessels as well as in the neuroparenchyma of retinal tissues from donors with DRD compared to non-diabetic donors (Figure 6l), consistent with the inner retinal dysfunction described above. Further highlighting the need to understand the role of these changes in the onset and progression of DRD, increased complement protein expression was one of the earliest identified perturbations in the vitreous, being detected in fluid from donors with diabetes without detectable DRD (Figure 6c–d).

## DISCUSSION

Diabetic retinal disease is a highly complex, progressive retinal condition that has been poorly understood due to difficulty in obtaining clinically relevant human samples for analysis. To date, severity and staging of diabetic retinopathy have exclusively focused on visible microvascular findings of leakage and underlying ischemia within the retina identified on clinical exam. To fill this knowledge gap, we performed integrated transcriptomic and proteomic analysis on vitreous and region-specific retinal samples from individuals with varying stages of DRD. Dysregulation of endothelial cell function and expression associated with known clinical vascular complications corresponded to disease severity in our dataset (Figure 2 and S5); however, despite angiogenesis and vascular remodeling being hallmarks of clinical DRD, these pathways were over-shadowed by evidence for whole retinal dysregulation through decreased cellular metabolism and neuronal and axonal impairment. We identified decreased expression of retinal function, specifically aerobic respiration and neuronal synaptic dynamics, and increased gliosis and neuroinflammation with activation of complement in the setting of progressive neurodegeneration in DRD. We performed computational analysis to identify specific cell-types driving these processes and confirmed expression changes of specific pathway targets within human retinal tissue samples validating the patterns identified by multiomic analysis. Our analysis provides primary evidence in human tissues to the: (1) involvement of the whole retinal neurovascular unit in DRD, (2) retinal region-specific mechanisms of DRD progression, (3) clinically-relevant molecular and cellular processes to identify novel biomarkers and therapeutic targets, and (4) potential and limitations of vitreous as a proxy of the retinal “state.”

Diabetes is considered a state of catabolism despite excess cellular fuel resulting in deficient or abnormal anabolic production of macromolecules (glycogen, lipids, proteins) and reduced ATP generation.^27^ We found decreased aerobic respiration via mitochondrial activity (i.e. ATP synthesis and electron transport chain function), which likely reflects decreased cellular activity and antioxidant capacity, localized to the macula in advancing disease in both proteomic and transcriptomic datasets (Figure 2).^28^ This regional finding matches with the known dependence of the macula on aerobic respiration and anabolic pathways compared to the peripheral retina, which is highly reliant on anaerobic respiration such as glycolysis,^29^ and corresponds with greater susceptibility of the macula to capillary non-perfusion characteristic of DRD. Additionally, downregulation of pathways associated with mitochondrial structure and energetics, ATP generation, and oxidative phosphorylation suggests an impaired response to oxidative injury,^30,31^ which can be highly deleterious to neuronal function due to baseline high metabolic activity of the neuroretina compared to other organs. Cellular metabolism is dependent on the intricate balance of redox signaling to manage both oxidative and reductive stress.^32–34^ Our data reveals an initial increase in expression of genes associated with aerobic respiration in the mild DRD stage (Figure 5a–b) accompanied by increased expression of proteins associated with ATP generation within the fovea (Figure 2d), which may signal to this cellular redox imbalance. Within these pathways, we found expression of electron transport chain Complex I NADH:ubiquinone oxidoreductase family decreased with advancing disease (Figure 2 and Figure S5a), which has been associated with dysregulated angiogenesis, axonal destruction, and demyelination.^24,33,35–39^ We also noted decreased expression of isocitrate dehydrogenase 3 alpha (IDH3A), a crucial enzyme within the tricarboxylic acid (TCA) cycle for NADPH generation and ATP production, with advancing DRD (Figures 5 and S5). Decreased enzymatic activity of IDH3A at birth is associated with retinal dystrophy^40^ and dysregulation of the TCA cycle in rods has been shown to result in photoreceptor outer segment defects in a mouse model suggesting a critical role for IDH3A in retinal metabolic function.^41^ With its primary expression in retinal neurons (Figure 5D), IDH3A reduction in tissues from diabetic donors coincides with decreased expression of synaptic vesicle trafficking and axonal transport proteins providing further evidence of diabetes-induced neurodegeneration. Supporting the overall disruption of the neurovascular unit, decreased expression of specific neuronal markers for synaptic function, such as Synaptotagmin-1 (SYT1) and Synaptosomal-Associated Protein, 25kDa (SNAP 25), concurred with increased neuroinflammation and glial activation within the macula in advanced DRD (Figures 5, S5 and S6). Cell-type deconvolution analysis further supported that abnormal metabolism within all retinal neurons, particularly in retinal ganglion cells, drives DRD disease progression in the fovea, while microglia and macroglia play a more preponderant role in the macula and periphery (Figure 3c–d).

Consistent with previous findings in animal models of diabetes, protein synthesis and protein stability as measured by pathways involved in transcription, mRNA processing, translation, and protein degradation were specifically affected with increasing disease severity. Interestingly, while transcriptomic and proteomic data showed a consistent enrichment of these pathways in the macula and fovea, proteomic data highlighted changes in post-transcriptional gene expression in mild DR (Figures 1k and 2). These changes may represent cellular re-organization and increasing stress response with disease. Upregulation of small heat shock proteins such as αA-crystallin have been shown to be neuroprotective in the stressed retinal neuron through anti-apoptotic function.^42–44^ Our work also revealed a specific dysregulation of retinal binding proteins (RBP), which are involved in post-transcriptional modulation of gene expression by affecting all stages of mRNA including pre-mRNA splicing, translational control, cleavage, polyadenylation, RNA stability and localization, and editing.^45^ Interestingly, expression of RBPs negatively correlate with diabetic retinopathy severity and associated vitreous inflammation consistent with the previously proposed protective potential of RBP3.^46,47^

Our dataset also highlights the important role of inflammation and activation of immunoprotective pathways in advancing DRD. Both the proteomic and transcriptomic datasets confirmed immune activation through antigen processing and presentation and complement. The blood retinal barrier, known to be disrupted in visually-threatening forms of DRD, is maintained by integrated function of endothelial cells, pro-angiogenic signaling, and supporting cellular structures such as the basement membrane and Müller glial cells. BRB disruptions and retinal ischemia have been associated with elevated complement pathway and activation of the innate immune system, especially in proliferative diabetic retinopathy.^48–50^ Our findings further corroborate increased complement activation suggesting a role for opsonization, inflammation, and even the formation of membrane attack complex (MAC) in retinal dysfunction. Furthermore, these changes not only localize to the macula and fovea but are present by proteomics even in donors with diabetes without clinical evidence of DRD, including within the vitreous (Figure 6).^51^ This and transcriptional level changes suggest both intraretinal induction of complement through either glial or microglial cell activation, as recruitment of infiltrating immune cells cannot be detected until late stages of the disease. Whether these downstream effects of complement are beneficial (i.e. removal of damaged cells and debris) or deleterious (i.e. inappropriate activation contributing to chronic inflammation, neurodegeneration and vascular damage) to human retinal function in DRD requires further mechanistic exploration.

Together the analysis suggests that abnormal metabolic function in neuronal cells such as retinal ganglion cells and photoreceptors, which are highly metabolically active and susceptible to oxidative stress,^52–54^ produces direct toxicity that drives neuroinflammation and along with microvascular damage lead to whole neurovascular retinal dysfunction (**Graphical Abstract**). Our mechanistic findings of neuronal dysfunction in early stages of disease support published clinical findings of rod and cone abnormalities seen by electroretinography and inner retinal architecture changes described in the absence of clinical DRD.^55–58^ Furthermore, neuroinflammation through microglial and Müller cell activation, corroborated by immunofluorescence, is consistent with prior reports as an early feature of DRD.^8,59,60^ Most interestingly, we identify activation of complement as a potentially targetable pathway in retinal tissue, which was mirrored within the diabetic vitreous compartment, including in those without clinical signs of DRD and appears to localize to areas of metabolic and axonal injury within the retina. With significant clinical challenges in obtaining retinal biopsies, the more accessible vitreous has become an appealing option to assess the local ocular environment and define treatment specificity. Multiple studies have confirmed an increased presence of inflammatory cytokines, such as interleukin-6 (IL-6), in the aqueous humor and vitreous in individuals with late stage DRD.^60–63^ Proteomic analysis performed on the vitreous from the same donors used for retinal analysis provided insight into the pathways unique to and shared with the underlying retinal tissue in varying stages of clinical disease. We were able to confirm complement and innate immune activation seen in the retina was reflected within the vitreous space (Figures S2 and Figure 4). While the role for complement inhibitors in diabetic retinopathy has not yet been clinically explored, our work suggests targeting complement activation and function intravitreally could be a viable approach to preserving the neurovascular unit and preventing subsequent disease progression such as neurodegeneration, ischemia or macular edema.

While human tissue work can provide meaningful insights into disease, retrospective studies performed on post-mortem samples do have important limitations to consider. Appropriate quality control measures and logistical frameworks allowed timely collection of these samples without significant tissue degradation. Our dataset did not include human retinal samples with severe proliferative changes or those undergoing treatment for DRD. While these samples could provide significant insight into vision-threatening complications of DRD, the functional changes observed within the retina may be confounded by common therapeutic strategies used such as vitrectomy, panretinal photocoagulation, and/or anti-vascular endothelial growth factor or steroid intravitreal therapy and thus were excluded from this study. Furthermore, our dataset is generated from bulk RNA and protein analysis rather than single cell analysis which precluded the identification of cellular differences. Cell-specific markers were used to confirm the presence of retinal cells and cellular deconvolution analysis was performed to overcome this challenge (Figures 3 and S3); however, our approach focused on the assessment of global gene expression changes. Further single-cell studies are necessary to confirm these findings and to understand the role of the retinal vasculature in the processes described here. Future work, such as that through the Mary Tyler Moore Vision Initiative, will establish frameworks to further integrate these types of datasets with single cell detail and other factors of DRD such as systemic health, visual function and patient-reported vision-related quality of life.

In summary, we present a comprehensive transcriptomic and proteomic dataset for varying clinical stages of DRD within the human retina and vitreous. The diverse cellular and functional changes described in this dataset confirms that diabetes affects the whole retina, not just the retinal blood vessels. By correlating both RNA and protein data from the same donor tissue followed by human tissue validation, we were able to confirm markers of neuroinflammation and decreased retinal metabolic and synaptic function that may serve as biomarkers in DRD staging and response. Furthermore, our analysis supports the need for a multi-pronged approach in treatment of DRD. These findings highlight the importance of a revised classification of DRD that better reflects the mechanisms occurring within the disease, specifically in early stages and targetable pathways within the vitreous compartment.

## Supplementary Material

1

This is a list of supplementary files associated with this preprint. Click to download.

- DataAccess.docx

## Figures and Tables

**Figure 1.**
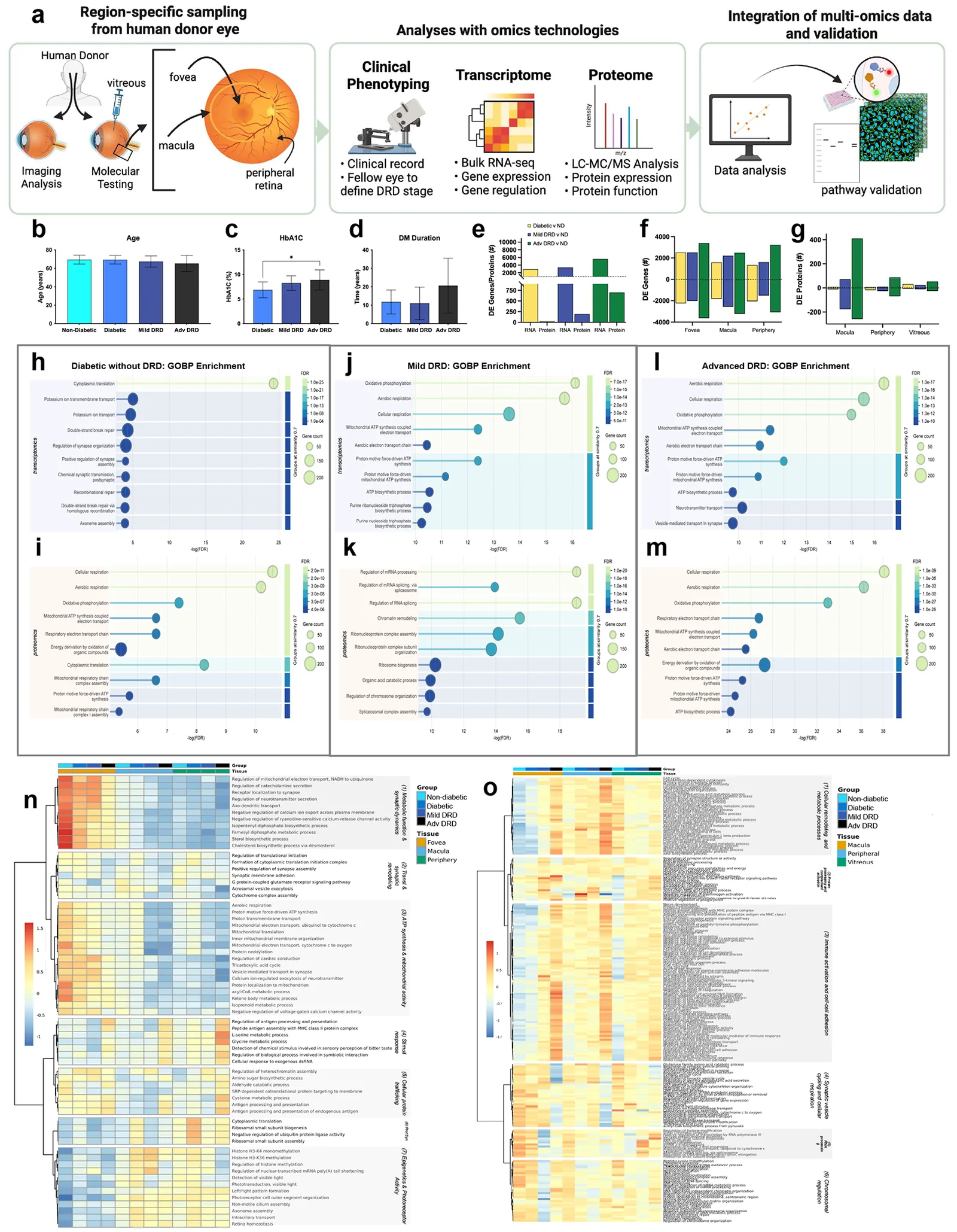
Multiomic approach to investigate human retinal tissue from individuals with varying stages of diabetic retinal disease (DRD). **(a)** Schematic overview of the data acquisition process for clinical phenotyping, proteomics, and RNA-sequencing on retinal tissue obtained from individuals with diabetes at different stages of diabetic retinal disease (DRD). Clinical phenotyping of donors with diabetes with and without DRD (non-diabetic, ND) used in the proteomic and transcriptomic analysis including distribution of underlying diabetes severity measured by (**b**) age (years), (**c**) hemoglobin A1C (HbA1C) (%), and (**d**) diabetes mellitus (DM) duration (years). (**e-g**) Number of differentially expressed (DE) (p<0.01) genes (RNA) and/or proteins are depicted for each comparison with down-regulated genes identified numerically below the x-axis in **f** and **g**. Gene ontology biological processes (GOBP) identified in (**h, j, l**) transcriptomic and (**i, k, m**) proteomic data by STRING analysis that were grouped by similarity of greater than 0.7 and sorted by −log(false discovery rate) when comparing (**h-i**) diabetes without DRD, (**j-k**) mild DRD, and (**l-m**) advanced DRD to those without diabetes. Disease effects on significant gene ontology biological processes (GOBP) were clustered into groups which are represented by heat map for (**n**) transcriptomic and (**o**) proteomic datasets by both disease state and location. Functional significance to clustered groups is indicated numerically.

**Figure 2.**
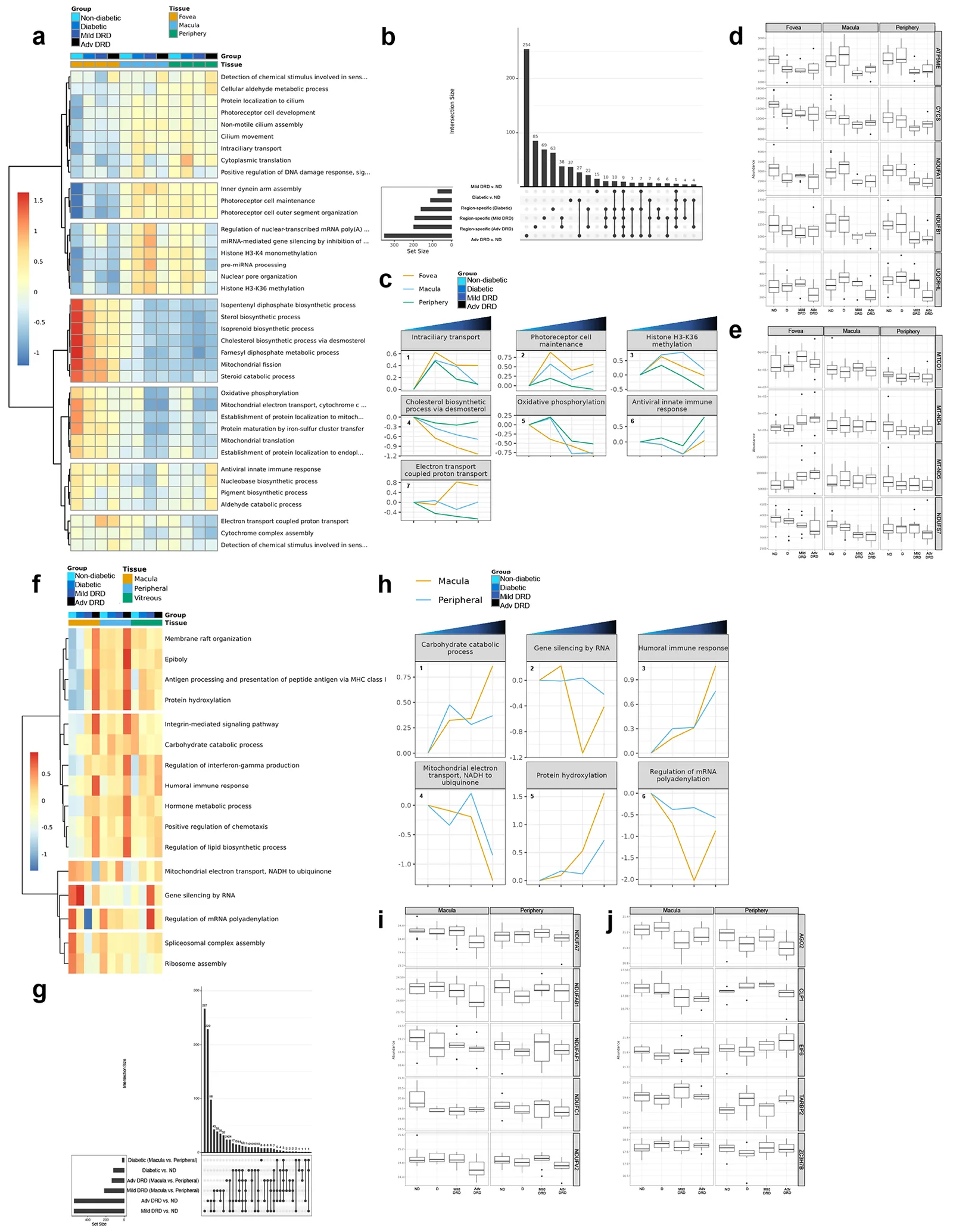
Identification of region-specific effects of advancing DRD within the human retina. Aggregated gene expression data for GO Biological Process (GOBP) categories that were enriched in genes with an interaction between disease stage and region effects were clustered into groups, which are represented by heat map for (**a**) transcriptomic and (**f**) proteomic datasets. Upset plot identifying the overlap between global and region-specific effects for (**b**) RNA and (**g**) protein for GO category enrichment. Overlap of GO processes are represented by number within the large bar graph and disease state in the adjoining dot plot. General sample comparisons are differentiated by region-specific comparisons of disease severity with location in parentheses. The set size of each comparison is indicated with a horizontal bar graph. Pathways identified as significantly different by region at one point in disease severity are highlighted as line plots in (**c**) transcriptomic and (**h**) proteomic data; these are drawn relative to non-diabetic samples for each disease stage as depicted by stage gradient from non-diabetic (light blue) to advanced DRD (black) from left to right. Boxplots, normalized to the have the same total counts, for each disease state representing genes identified with the largest interaction effect when comparing region to disease severity for GOBP (**d**) oxidative phosphorylation (GO:0006119) and (**e**) electron transport coupled protein transport (GO: 0015990) within the transcriptomic dataset and for GOBP (**i**) mitochondrial electron transport, NADH to ubiquinone (GO:0006120) and (**j**) gene silencing by RNA (GO:0031047) within the proteomic dataset.

**Figure 3.**
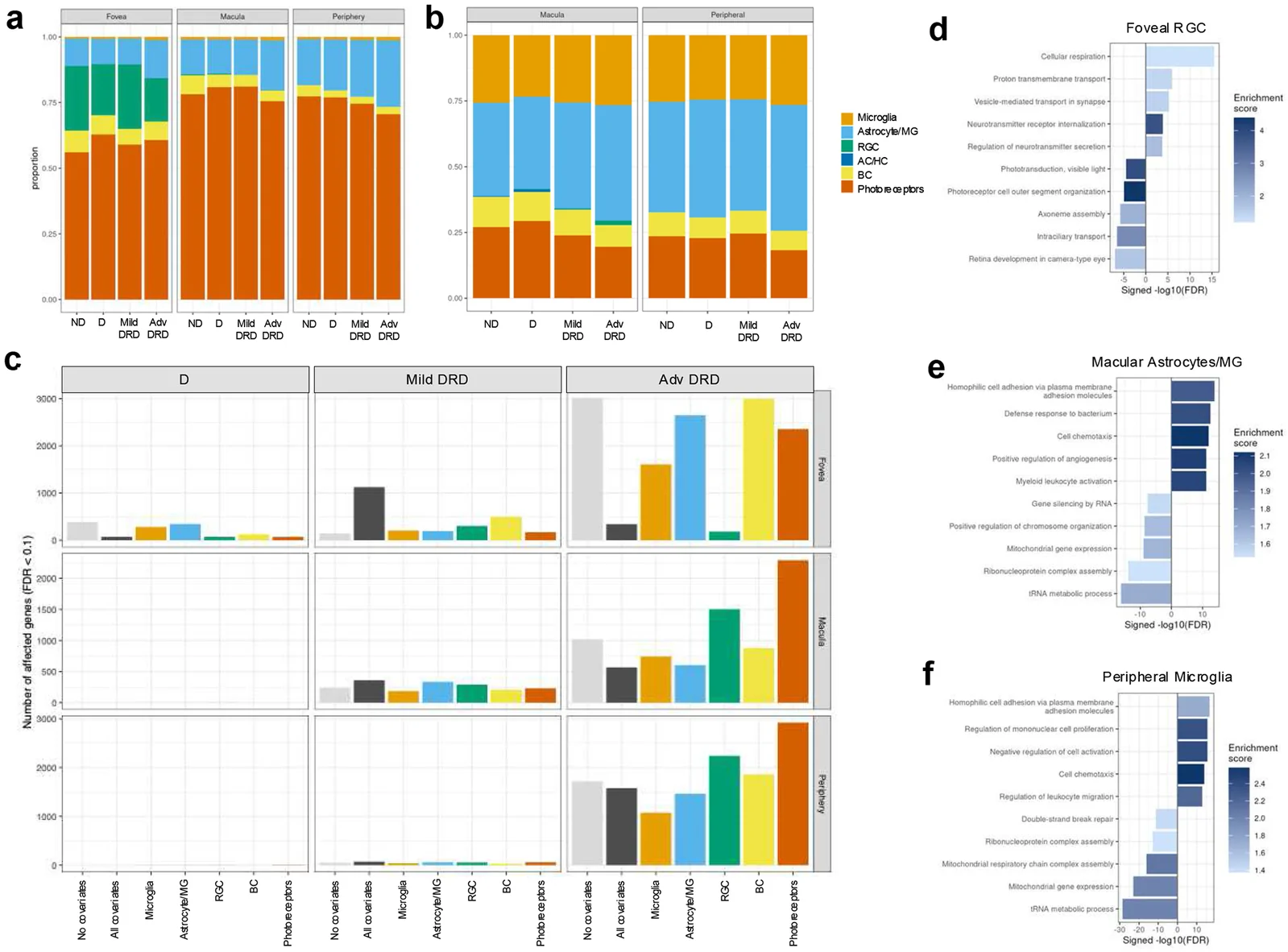
Whole retinal neuroglial unit dysfunction is present in advanced DRD. Cell-type deconvolution of bulk RNA samples were performed with AutoGeneS and mean cell proportions for retinal cell type for each disease and region combination are shown for (**a**) transcriptomics and (**b**) proteomics. (**c**) The number of genes affected (FDR < 0.1) in a linear model of disease with no additional covariates, all cell-type proportions as covariates or individual cell type proportions separately as covariates. (**d-f**) Functional enrichment analysis results are shown for genes associated with individual cell type fractions in a model of gene expression comparing advanced DRD vs non-diabetic samples within regions: (**d**) genes associated with RGC in the fovea, (**e**) genes associated with Müller glial cells and astrocytes in the macula or (**f**) genes associated with microglia in the periphery. Abbreviations: AC: Amacrine cell; BC: Bipolar cell; HC: Horizontal cell; MG: Müller Glia; RGC: Retinal ganglion cell.

**Figure 4.**
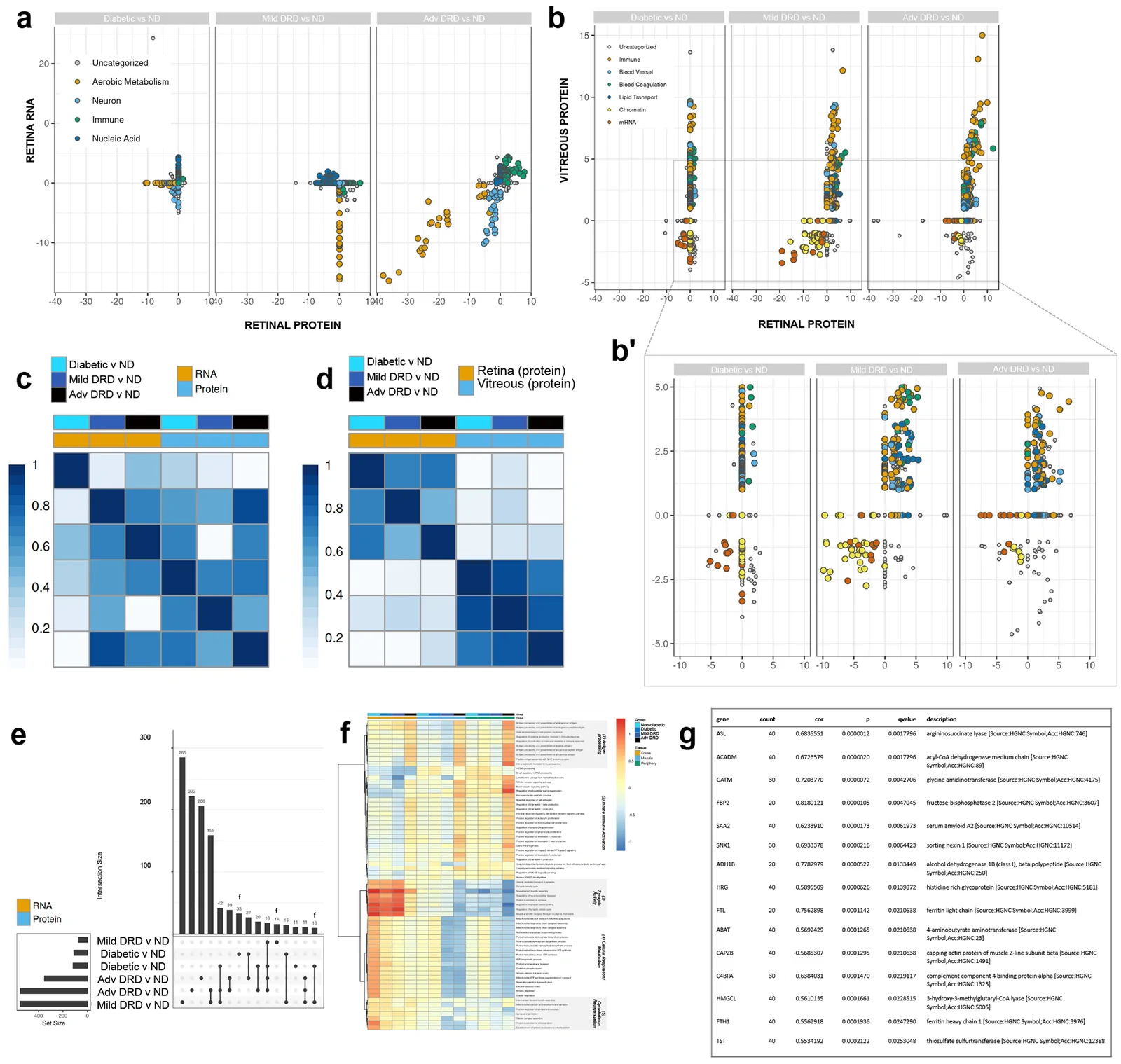
Correlation of biological processes identified between the transcriptomic and proteomic datasets. Scatter plots representing the overlap of gene ontology biological processes (GOBP) enrichment between (**a**) retinal RNA and protein analyses and (**b**) retinal protein to vitreous protein when comparing diabetic, mild DRD, and advanced DRD to non-diabetic donors. A detailed version of the markers identified by the box in **b** is enlarged in **b’**. Heatmap of correlation (R^2^) between differential expression of GOBP from RNA and protein obtained from the retina (**c**) and retinal protein and vitreous protein (**d**). (**e**) Upset plot of the effect of disease severity on GO processes for analysis of the RNA and protein data. Overlaps of GO processes are represented by number within the large bar graph and disease state in the adjoining dot plot with set size depicted by the horizontal bar graph. RNA sample comparisons are indicated with orange highlight and protein with blue highlight. (**f**) Heatmap of intersecting GO processes identifying pathways enriched in advanced DRD by both transcriptomic and proteomic analysis as identified in **e** shown with respect to RNA. (**g**) Top correlating genes identified between the retina and vitreous protein.

**Figure 5.**
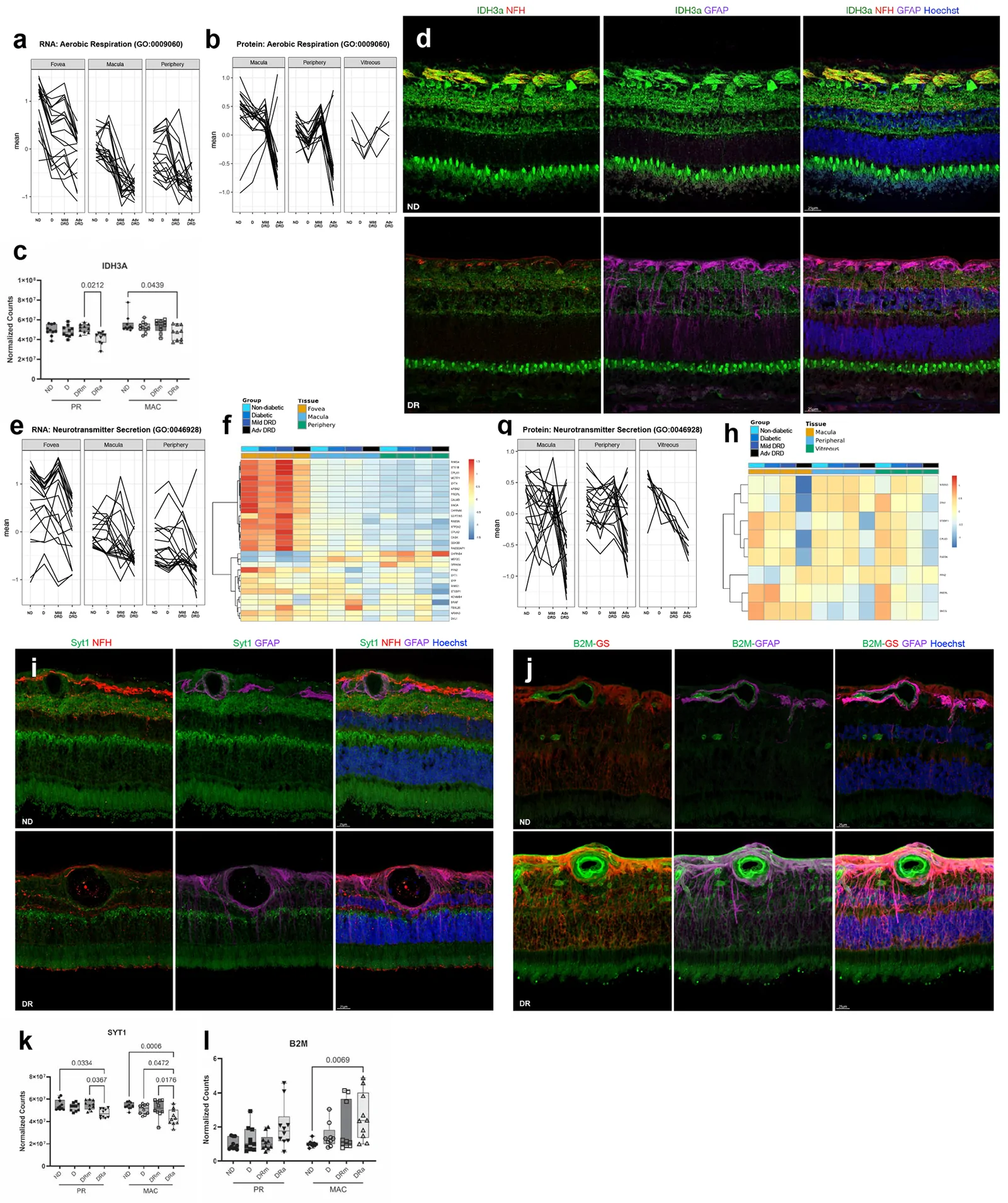
Decreased metabolic and axonal function and increased neural gliosis correlate to advancing macular DRD in the human retina. Line plots of normalized (**a**) RNA and (**b**) protein abundances for top 20 affected genes in gene ontology biological process (GOBP) aerobic respiration (GO:0009060). (**c**) Normalized counts for protein expression of isocitrate dehydrogenase 3 alpha (IDH3A) measured by Western Blot analysis in human tissue from non-diabetic (ND), diabetic without DRD (D), mild DRD (DRm), and advanced DRD (DRa) for peripheral retina (PR) and macula (MAC) with associated p-value comparisons. (**d**) Immunohistochemical analysis of non-diabetic human retina sample (ND) compared to advanced DRD samples (DR) with associated stain for metabolic function (isocitrate dehydrogenase 3 alpha (IDH3A). Line plots of normalized (**e**) RNA and (**g**) protein abundances for top 20 affected genes in GOBP regulation of neurotransmitter secretion (GO:0046928) with associated heatmap for the top (**f**) genes and (**h**) proteins for each respective dataset. (**i-j**) Immunohistochemical analysis of non-diabetic human retina samples (ND) compared to advanced DRD samples (DR) are shown with neuronal markers (Synaptotagmin-1 (SYT1), glial fibrillary associated protein (GFAP), neurofilament protein H (NFH), and Müller glial cell marker glutamine synthetase (GS) with extracellular matrix protein (beta-2 microglobulin (B2M)) and nuclear marker Hoeschst. (**k**) Synaptotagmin-1 (SYT1) and (**l**) beta-2 microglobulin (B2M) measured by Western Blot analysis. Abbreviations: MAC: Macula: PR: Peripheral retina

**Figure 6.**
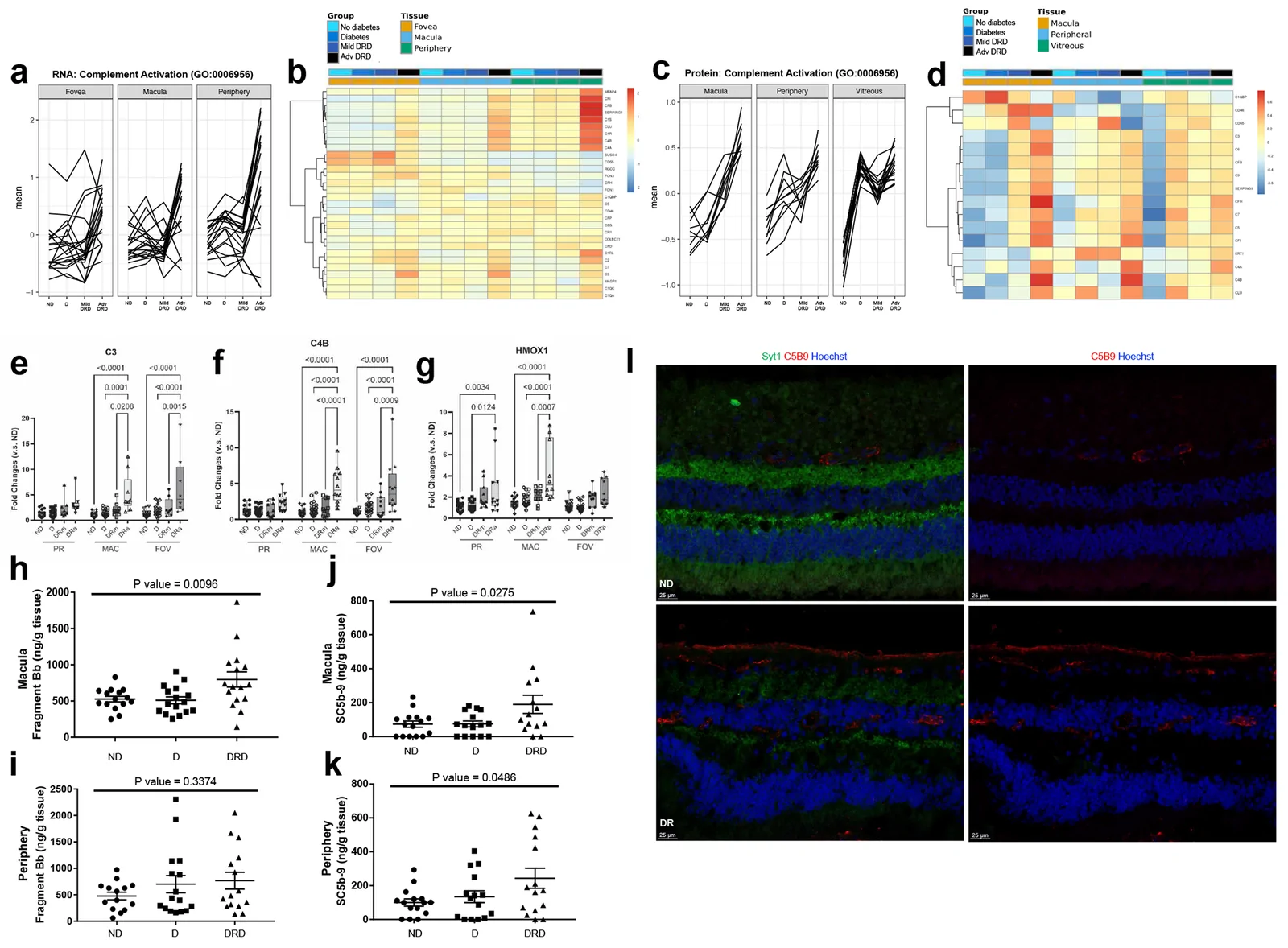
Increased complement activation correlates with DRD severity in the human retina in a region-specific manner. Line plots of normalized (**a**) RNA and (**c**) protein abundances for top 20 affected genes in complement activation gene ontology biological process (GO:0006956). Heatmap represents the top (**b**) genes and (**d**) proteins for each respective dataset. Fold change of disease states (diabetic without DRD (D), mild DRD (DRm), and advanced DRD (DRa)) compared to non-diabetic (ND) of complement proteins (**e**) C3 and (**f**) C4B and (**g**) heme oxygenase 1 (HMOX1) were confirmed in human retinal tissue by qPCR. ELISA was performed on human retinal samples from non-diabetic (ND, n= 14-15), diabetic (D, n=15-16), and DRD (n=14-16) for expression of (**h, i**) Fragment Bb and (**j, k**) membrane attack complex (MAC) SC5b-9 in the macula and peripheral retina. (**l**) Expression of MAC components were present in non-proliferative DRD samples and advanced DRD samples compared to non-diabetic controls. Abbreviations: PR: Peripheral retina; MAC: macula; FOV: fovea

**Table 1.** Summary of Demographic Variables for Donors included in Proteomic and Transcriptomic Analysis.

Donor Group	N	Age (years)	Sex (F/M)	HbA1c, % (#)	Diabetes Type (1/2/UK)	Disease Duration (years)	# of medical co-morbidities at time of death
No diabetes (ND)	21	69.4±4.7	11/10	N/A	N/A	N/A	2.9±1.7
Diabetes without DRD	23	69.3±4.6	10/13	6.9±1.6 (17)	0/22/1	11.7±6.3 (15)	3.9±2.1
Mild DRD	11	67.5±5.8	3/8	8.3±1.4 (8)	0/11/0	11 ±8.0 (6)	5.5±1.7
Advanced DRD	11	65.3±8.4	2/9	8.9±2.0 (4)	2/8/1	20.6±13.9 (8)	3.7±2.0

Abbreviations: DRD, Diabetic Retinal Disease; N, sample size; N/A, not available; F, female; M, male; HbA1c, Hemoglobin A1c; UK, unknown. Averages are displayed with mean ± standard deviation when applicable. All collected samples were described as Caucasian in redacted medical charts. DR stage was determined by available ocular history and evaluation of the contralateral eye. The average diabetes duration and HbA1c were calculated for the donors where this information was available (n of available donors in parenthesis).

## Data Availability

The RNA-seq data generated in this study have been deposited in the NCBI Gene Expression Omnibus (GEO) under accession number GSE349973. The mass spectrometry proteomics data have been deposited to the ProteomeXchange Consortium via Massive with the dataset identifier PXD085589.
