# Supplementary material for: Multiomic analysis of human diabetic retinal disease reveals severity and region-specific impairment of the retinal neurovascular unit
Source: Res Sq. 2026 Oct 30:rs.3.rs-11394372. Preprint. [Version 1] doi: 10.21203/rs.3.rs-11394372/v1 (PMC13734275; doi:10.21203/rs.3.rs-11394372/v1)
Supplement: Supplementary file 1 [file NIHPPrs11394372v1-supplement-1.pdf]

# Supplemental Figure 1

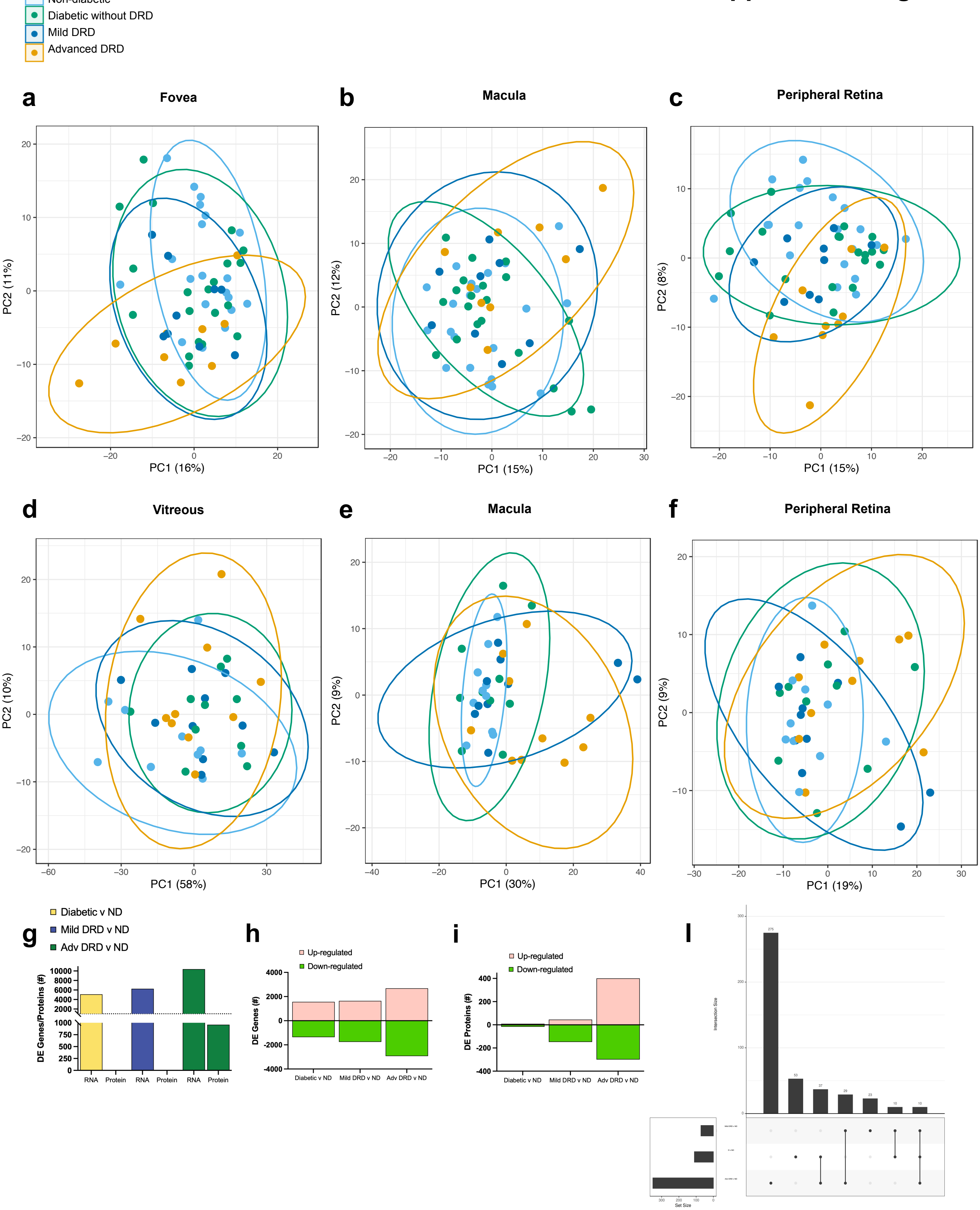

**Supplemental Figure 1. Multiomic approach to human retinal tissue from individuals with diabetes.** Principal component analysis (PCA) plots of samples from each disease cohort for (**a-c**) RNA and (**d-f**) protein using the top 500 genes or proteins respectively**.** (**g-i**) Number of differentially expressed (DE) (FDR<1) genes (RNA) and/or proteins are depicted for each comparison. The total number of differentially expressed up- and down-regulated (**h**) genes and (**i**) proteins identified by p-value <0.01. (**l**) Upset plot of direct effects of disease severity on gene ontology (GO) processes for analysis of the RNA data. Overlap of GO processes are represented by number within the large bar graph and disease state in the adjoining dot plot with set size depicted by horizontal bar graph.

# Supplemental Figure 2

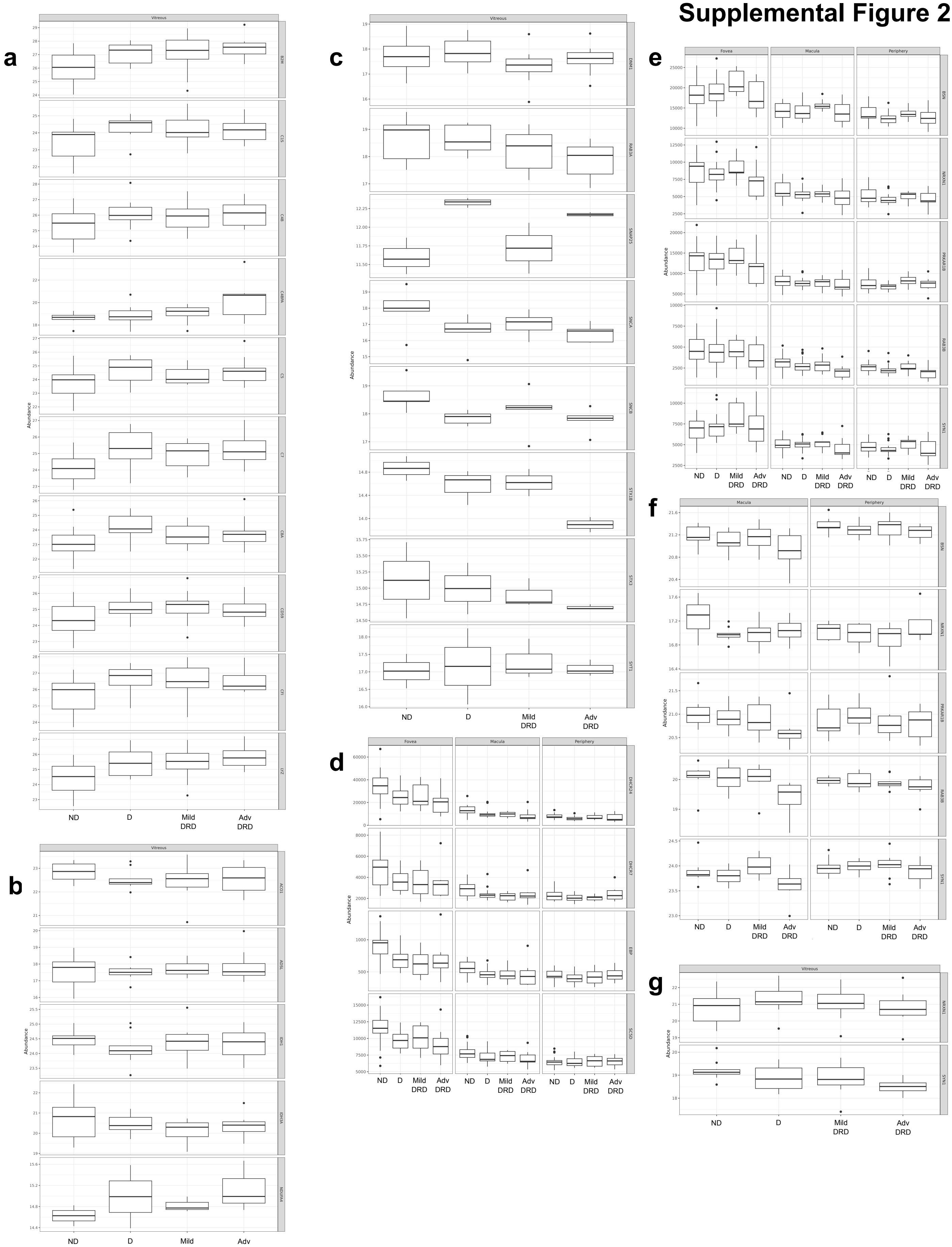

**Supplemental Figure 2**. **Identification of biological processes within the human vitreous and retina affected by DRD in a region-specific manner.** Box plots for gene products associated with (a) complement immune response, (b) oxidative phosphorylation, and (c) neurodegeneration within the vitreous. (d) Box plots for each disease state representing genes identified with the largest interaction effect when comparing region to disease severity for GOBP cholesterol biosynthetic process via desmosterol (GO:0033489). Boxplots for the top affected global genes for (e) RNA and (f-g) protein in the GOBP regulation of synaptic vesicle cycle (GO:0016188) are listed by region. For all boxplots, samples were normalized to have the same total counts.

# Supplemental Figure 3

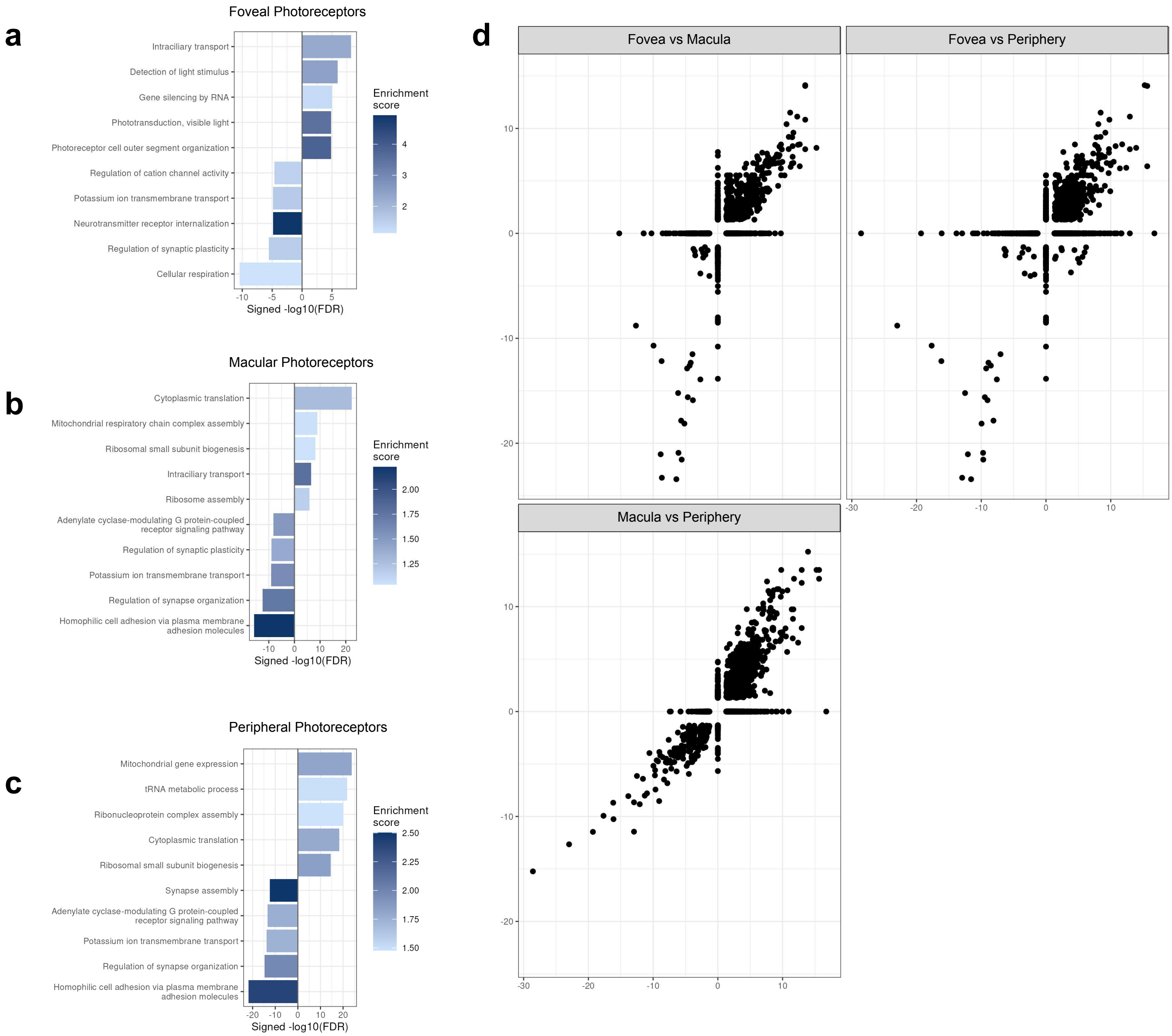

**Supplemental Figure 3. Whole retinal neuroglial unit dysfunction is present in advanced DRD.** (**a-c**) Functional enrichment results for gene associated with photoreceptors comparing advanced DRD to ND donors in (**a**) fovea, (**b**) macula, and (**c**) periphery within the transcriptomic data. (**d**) Comparison GOBP enrichment scores for the proportion of microglia-associated genes between fovea, macula and periphery in advanced DRD compared to non-diabetic donors (ND) for transcriptomic dataset. Signed –log10(FDR) is represented on the x and y-axes.

# Supplemental Figure 4

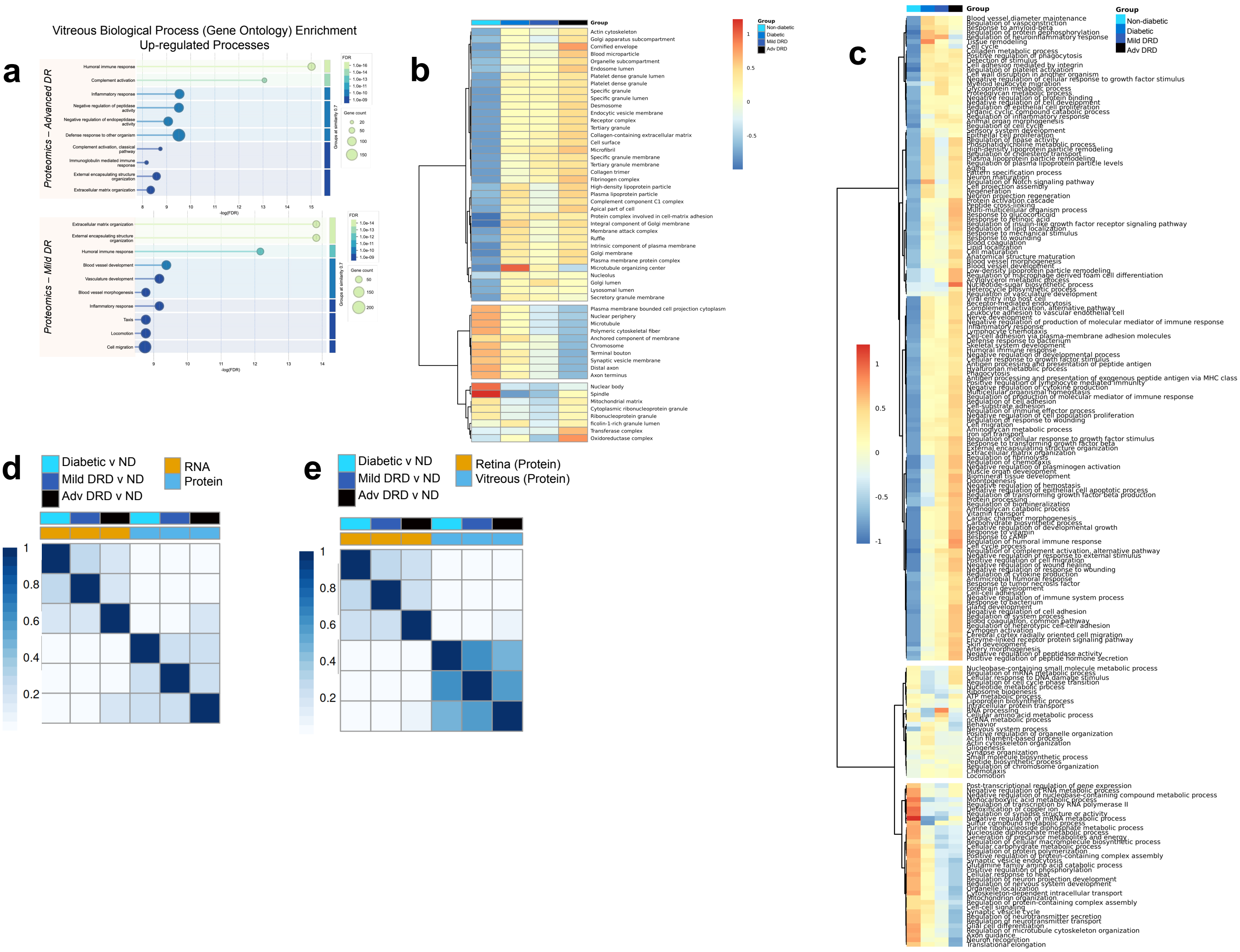

**Supplemental Figure 4. Identification of biological processes associated with DRD severity in the vitreous compartment.** (**a**) Up-regulated biological processes identified in proteomic analysis of the vitreous compartment for in advanced DRD and mild DRD to those without diabetes. Heatmap representing the gene ontology (**b**) cellular component and (**c**) biological processes by disease state on vitreous proteins expression. Heatmap of correlation ($R^2$) maps are shown for the overlap of genes between the retinal (**d**) RNA and (**e**) protein to vitreous protein.

# Supplemental Figure 5

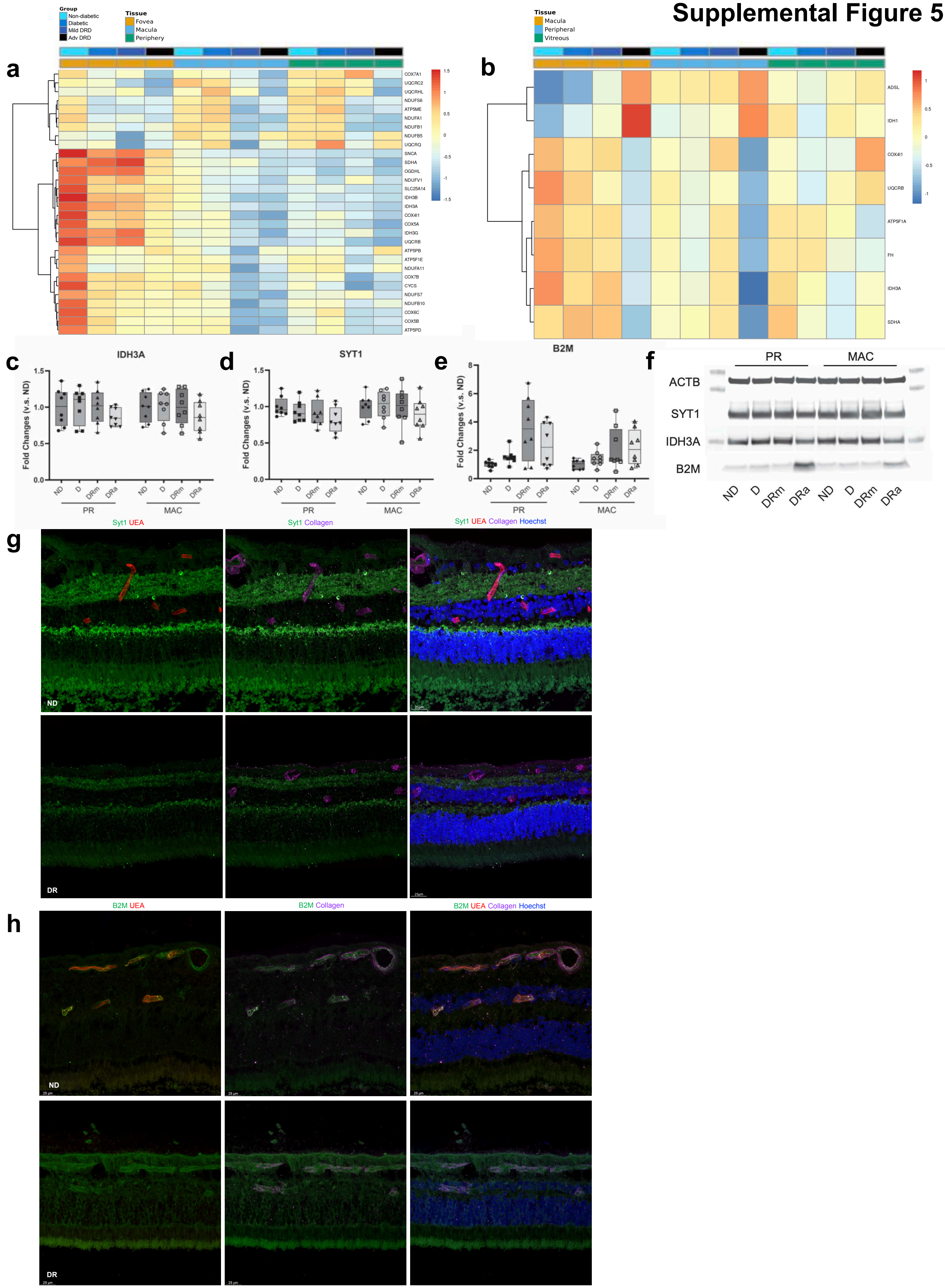

**Supplemental Figure 5**. **Decreased metabolic and axonal function correlate to advancing macular DRD in the human retina.** Heatmap represents the top (**a**) genes and (**b**) proteins for GOBP Aerobic respiration (GO:0009060) associated with main **Figure 5**. Fold change compared to ND of (**c**) isocitrate dehydrogenase 3 alpha (IDH3A), (**d**) Synaptotagmin-1 (SYT1), and (**e**) beta-2 microglobulin (B2M) measured by western blot analysis compared to control marker (**f**) actin-B (ACTB). (**g-h**) Immunohistochemical analysis of non-diabetic human macular retina samples (ND) compared to the macula in advanced DRD (Adv DRD) are shown with associated stains for neuronal markers (Synaptosomal-Associated Protein, 25kDa (SNAP 25), glial fibrillary associated protein (GFAP), and neurofilament protein H (NFH)), and markers of vascular structures (collagen, Ulex europaeus agglutinin-1 (UEA), and beta-2 microglobulin (B2M)). Abbreviations: MAC: Macula: PR: Peripheral retina

# Supplemental Figure 6

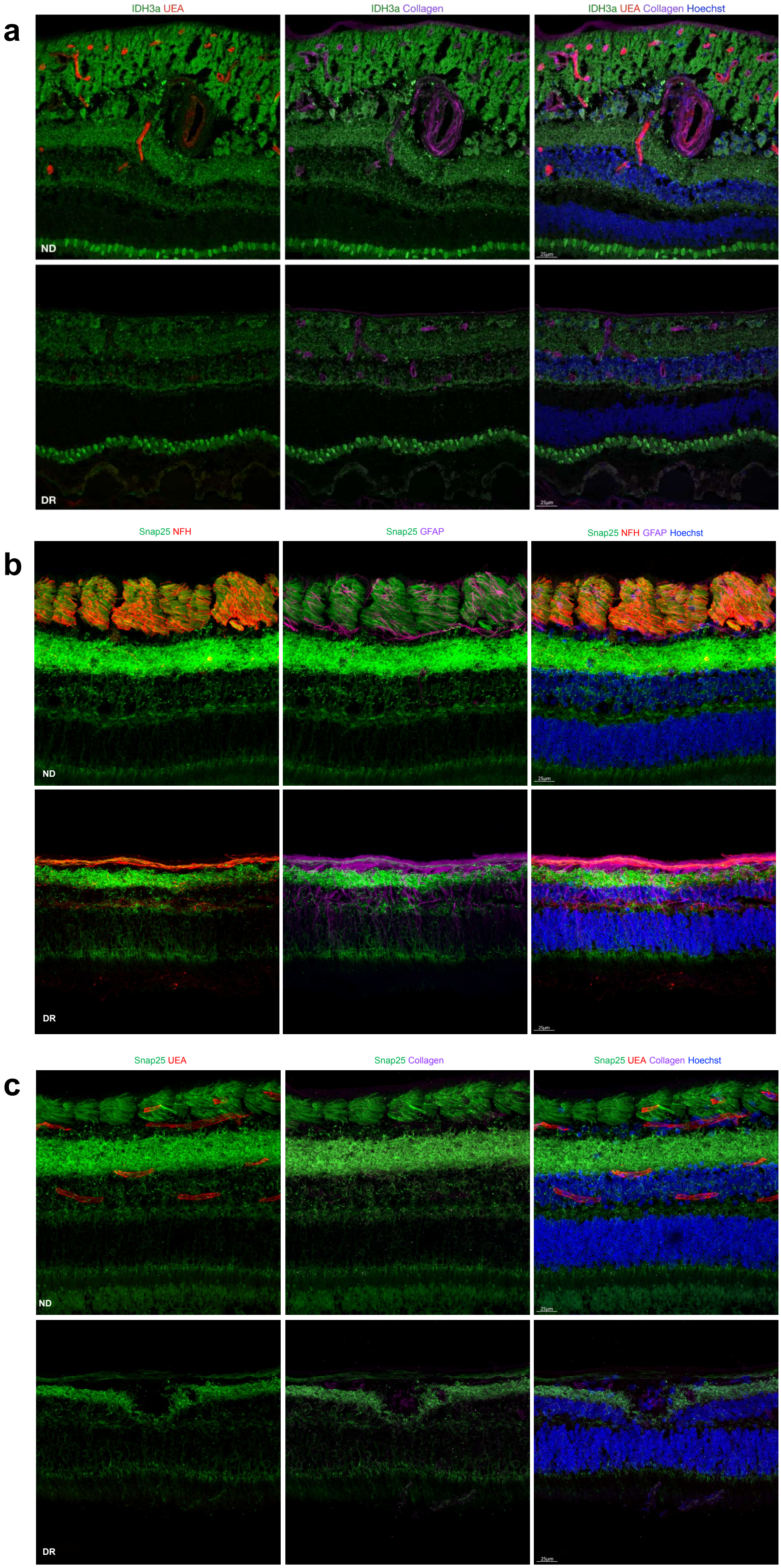

**Supplemental Figure 6. Decreased metabolic and axonal function correlate to advancing macular DRD in the human retina. (a-c)** Immunohistochemical analysis of non-diabetic human retina samples (ND) compared to advanced DRD samples (DR) are shown with associated stains for isocitrate dehydrogenase 3 alpha (IDH3A), Synaptotagmin-1 (SYT1), collagen, Synaptosomal-Associated Protein, 25kDa (SNAP 25), and Ulex europaeus agglutinin-1 (UEA).

# Supplemental Table 1

| Donor Group | N | Age (years) | Sex (F/M) | HbA1c (%) | Diabetes Type (1/2/UK) | Disease Duration (years) | # of medical co-morbidities at time of death |
|---|---|---|---|---|---|---|---|
| No diabetes | 21 | 69.4±4.7 | 11/10 | N/A | N/A | N/A | 2.9±1.7 |
| Diabetes without DR | 20 | 68.9±4.6 | 9/11 | 6.8±1.6 (15) | 0/19/1 | 11.9±6.5 (13) | 3.8±2.2 |
| Mild DR | 9 | 68.2±6.1 | 2/7 | 8.0±1.2 (7) | 0/9/0 | 11.0±8.8 (5) | 5.4±1.8 |
| Moderate-Severe DR | 9 | 64.2±8.7 | 2/7 | 8.9±2.0 (3) | 2/6/1 | 23.3±12.8 (7) | 3.4±1.1 |

**Supplemental Table 1. Summary of Demographic Variables for Donors included in Transcriptomic Analysis.** Abbreviations: DR, Diabetic Retinopathy; N, sample size; N/A, not available; F, female; M, male; HbA1c, Hemoglobin A1c; UK, unknown. Averages are displayed with mean ± standard deviation when applicable. All collected samples were described as Caucasian in redacted medical charts. DR stage was determined by available ocular history and evaluation of the contralateral eye. The average diabetes duration and HbA1c were calculated for the donors where this information was available (n of available donors in parenthesis).

# Supplemental Table 2

| Donor Group | N | Age (years) | Sex (F/M) | HbA1c (%) | Diabetes Type (1/2/ UK) | Disease Duration (years) | # of medical co-morbidities at time of death |
|---|---|---|---|---|---|---|---|
| No diabetes | 10 | 68.0±4.3 | 4/6 | N/A | N/A | N/A | 2.8±1.9 |
| Diabetes without DR | 10 | 68.7±5.2 | 4/6 | 7.1±1.6 (6) | 0/9/1 | 12±3.6 (6) | 3.5±1.7 |
| Mild DR | 10 | 67.9±5.9 | 3/7 | 8.1±1.4 (7) | 0/10/0 | 11±8.0 (6) | 5.5±1.7 |
| Moderate-Severe DR | 10 | 64.8±8.7 | 2/8 | 8.9±1.8 (4) | 1/8/1 | 16.3±8.4 (7) | 3.9±2.2 |

**Supplemental Table 2. Summary of Demographic Variables for Donors included in Proteomic Analysis.** Abbreviations: DR, Diabetic Retinopathy; N, sample size; N/A, not available; F, female; M, male; HbA1c, Hemoglobin A1c; UK, unknown. Averages are displayed with mean ± standard deviation when applicable. All collected samples were described as Caucasian in redacted medical charts. DR stage was determined by available ocular history and evaluation of the contralateral eye. The average diabetes duration and HbA1c were calculated for the donors where this information was available (n of available donors in parenthesis).

# Supplemental Table 3

| Gene Ontology Biological Process (Description) | Abbreviation associated with panel d in Figure 2 |
|---|---|
| **Mitochondrial translation** | Mitochondrial translation |
| **Aerobic respiration** | Aerobic respiration |
| **Regulation of mitochondrial electron transport, NADH to ubiquinone** | Regulation of mito. electron transport |
| **Sterol biosynthetic process** | Sterol biosynthetic process |
| **Isopentenyl diphosphate biosynthetic process** | Isopentenyl diphosphate biosynthetic process |
| **Farnesyl diphosphate metabolic process** | Farnesyl diphosphate metabolic process |
| **Cholesterol biosynthetic process via desmosterol** | Cholesterol biosynthetic process via desmosterol |
| **Methylglyoxal metabolic process** | Methylglyoxal metabolic process |
| **Histone H3-K4 monomethylation** | Histone H3-K4 monomethylation |
| **Axoneme assembly** | Axoneme assembly |
| **Negative regulation of calcium ion export across plasma membrane** | Neg. regulation of calcium ion export |
| **Rhombomere development** | Rhombomere development |
| **Cytoplasmic translation** | Cytoplasmic translation |
| **Negative regulation of ubiquitin protein ligase activity** | Neg. regulation of ubiquitin protein ligase activity |
| **Antigen processing and presentation of endogenous peptide antigen** | Antigen processing and presentation |
| **Acrosomal vesicle exocytosis** | Acrosomal vesicle exocytosis |

| Gene Ontology Biological Process (Description) | Abbreviation associated with panel h in Figure 2 |
|---|---|
| **Mitochondrial ATP synthesis coupled electron transport** | Mito. ATP synthesis coupled electron transport |
| **Antigen processing and presentation of peptide antigen via MHC class I** | Antigen proc.and pres. of peptide via MHC class I |
| **Regulation of membrane repolarization during action potential** | Reg. of memb. repolar. during action potential |
| **Regulation of synaptic vesicle cycle** | Regulation of synaptic vesicle cycle |
| **Mesodermal cell differentiation** | Mesodermal cell differentiation |
| **Endothelial cell morphogenesis** | Endothelial cell morphogenesis |
| **Negative regulation of epithelial cell differentiation** | Neg. regulation of epithelial cell differentiation |
| **Membrane raft organization** | Membrane raft organization |
| **RISC complex assembly** | RISC complex assembly |
| **Regulation of mRNA polyadenylation** | Regulation of mRNA polyadenylation |
| **Negative regulation of alcohol biosynthetic process** | Negative regulation of alcohol biosynthetic process |
| **Establishment of centrosome localization** | Establishment of centrosome localization |
| **Protein hydroxylation** | Protein hydroxylation |
| **Histone H3-K27 trimethylation** | Histone H3-K27 trimethylation |
| **Transepithelial chloride transport** | Transepithelial chloride transport |

**Supplemental Table 3. Gene Ontology Biological Processes Associated with Main Figure 2.**
